# Photochemically active DNA-intercalating ruthenium and related complexes – insights by combining crystallography and transient spectroscopy

**DOI:** 10.1039/c7sc01070b

**Published:** 2017-04-12

**Authors:** Christine J. Cardin, John M. Kelly, Susan J. Quinn

**Affiliations:** a School of Chemistry , University of Reading , Whiteknights , RG6 6AD , UK . Email: c.j.cardin@reading.ac.uk; b School of Chemistry , Trinity College Dublin , Dublin 2 , Ireland . Email: jmkelly@tcd.ie; c School of Chemistry , University College Dublin , Belfield , Dublin 4 , Ireland . Email: susan.quinn@ucd.ie

## Abstract

Recent research on the study of the interaction of ruthenium polypyridyl compounds and defined sequence nucleic acids is reviewed. Particular emphasis is paid to complexes [Ru(LL)_2_(Int)]^2+^ containing potentially intercalating ligands (Int) such as dipyridophenazine (dppz), which are known to display light-switching or photo-oxidising behaviour, depending on the nature of the ancillary ligands. X-ray crystallography has made a key contribution to our understanding, and the first complete survey of structural results is presented. These include sequence, enantiomeric, substituent and structural specificities. The use of ultrafast transient spectroscopic methods to probe the ultrafast processes for complexes such as [Ru(TAP)_2_(dppz)]^2+^ and [Ru(phen)_2_(dppz)]^2+^ when bound to mixed sequence oligonucleotides are reviewed with particular attention being paid to the complementary advantages of transient (visible) absorption and time-resolved (mid) infra-red techniques to probe spectral changes in the metal complex and in the nucleic acid. The observed photophysical properties are considered in light of the structural information obtained from X-ray crystallography. In solution, metal complexes can be expected to bind at more than one DNA step, so that a perfect correlation of the photophysical properties and factors such as the orientation or penetration of the ligand into the intercalation pocket should not be expected. This difficulty can be obviated by carrying out TRIR studies in the crystals. Dppz complexes also undergo insertion, especially with mismatched sequences. Future areas for study such as those involving non-canonical forms of DNA, such as G-quadruplexes or i-motifs are also briefly considered.

## Abstract 2

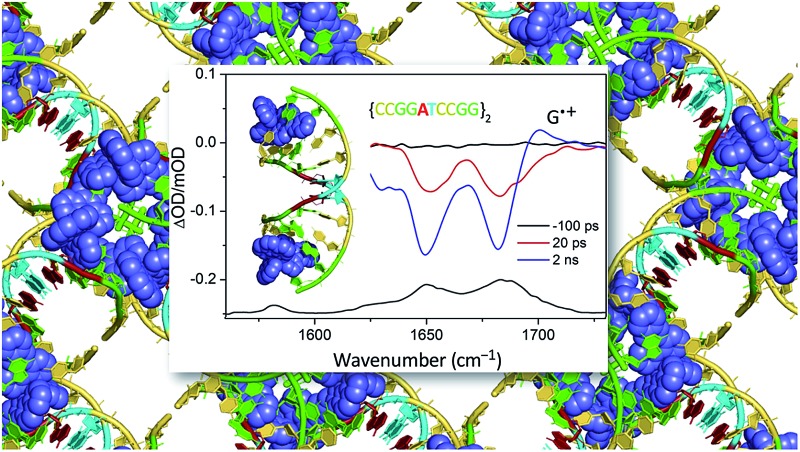
Recent research on the study of the interaction of ruthenium polypyridyl compounds and defined sequence nucleic acids is reviewed.

## 1. Introduction

Since the 1980s^1–3^ the study of DNA-binding ruthenium polypyridyls has afforded a rich vein of insight into the relationship between biomolecular structure and photophysical dynamics. These complexes have been effectively employed both to signal and image the presence of DNA secondary structure and to trigger photoinduced processes.^4,5^ Ruthenium polypyridyls make excellent photosensitisers, as their optical, photophysical and electrochemical properties can be readily tuned by changing the ligands around the central metal ion.^6^ Most complexes absorb in the visible, making their excitation by a range of light sources very convenient. The excited states (in most cases a triplet metal-to-ligand charge transfer, ^3^MLCT, state) are usually relatively long-lived (hundreds of nanoseconds) and they are often strong oxidizing and reducing agents. Such properties generated significant interest in solar energy applications, such as light induced dissociation of water, and this was the primary reason for the rapid expansion in the study of the photochemistry and photophysics of these compounds in the 1970s and 1980s. Indeed, such studies have been reinvigorated in the last decade or so with the realisation that there is a need to devise new routes to solar-generated fuels.^7^

Early pioneering studies of the biological applications of the ruthenium complexes were carried out in Australia by Dwyer and his collaborators.^8^ However, in general, this early work did not consider the fact that complexes with three bidentate ligands exist as enantiomers. The first work on enantiomeric specificity of ruthenium complexes, following on early observations with iron complexes,^9^ was by the Barton group, who studied the interaction of the Δ and Λ enantiomers of [Ru(phen)_3_]^2+^ with double-stranded DNA.^2,10^ This showed that indeed, as might be intuitively expected, the delta enantiomer bound more strongly to B-DNA than the lambda compound did. The same authors also proposed that [Ru(phen)_3_]^2+^ binds to B-DNA by insertion of a phenanthroline group between the base-pairs of the polynucleotide (*i.e.* intercalation), a mode of binding that did not occur for [Ru(bpy)_3_]^2+^. Intercalation, which was originally proposed by Lerman for planar heteroaromatics,^11^ causes a lengthening and unwinding of the double helix.

The proposal of intercalation by [Ru(phen)_3_]^2+^ was further supported by Kelly *et al.*, using topoisomerase experiments to demonstrate that the expected unwinding occurred for the racemic complex.^3^ However, later detailed spectroscopic and hydrodynamic experiments by Chaires and co-workers showed that the binding of [Ru(phen)_3_]^2+^ was through semi-intercalation – a process which caused a kinking of the DNA with a consequent effect on the viscosity, especially marked for the Δ-enantiomer.^12^ This binding mode leads to much stronger association of [Ru(phen)_3_]^2+^ than of [Ru(bpy)_3_]^2+^, a feature which means that the latter complex is readily displaced by increasing the ionic strength of the medium. The comparative binding modes of [Ru(phen)_3_]^2+^ and [Ru(bpy)_3_]^2+^ have been studied in detail using linear and circular dichroism by Lincoln and Nordén.^13^

**Fig. 1:**
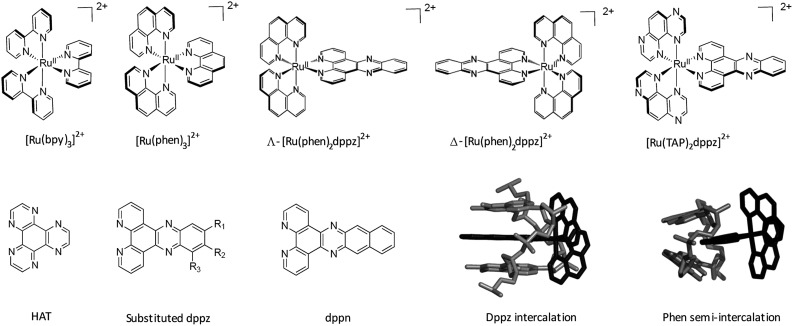
Overview of the structures of [Ru(LL)_3_]^2+^ and examples of extended ligands with illustrated intercalation modes.

A clear way to increase the strength of binding interactions through intercalation is to incorporate a more extended heteroaromatic ligand. One early example of this is the complex [Ru(bpy)_2_HAT]^2+^ which binds much more strongly and also shows more intense luminescence when DNA-bound than does the parent [Ru(bpy)_3_]^2+^ complex.^14^ However, the key advance that allowed highly effective intercalation was the incorporation of a dipyrido[3,2-*a*:2′,3′-*c*]phenazine (dppz) ligand. The first such compound, reported by the Barton group, was [Ru(bpy)_2_dppz]^2+^, which was found to bind strongly through intercalation.^15^ Other approaches to improve the strength of binding interaction have involved the use of appended or tethered intercalating ligands such as pyrene and naphthalimide groups^16^ as well as binuclear ruthenium polypyridyl systems.^17^ However, for the purposes of this review we will consider the interactions of mononuclear species with simple polypyridyl scaffolds, see Fig. 1.

A striking property of [Ru(bpy)_2_dppz]^2^ is that it is non-emissive in aqueous solution but luminesces strongly when it binds to DNA.^15^ This light-switching property is a consequence of the formation in water of a rapidly-deactivating ‘dark’ excited state, distinct from the ‘bright’ species present in non-aqueous environments.^4c,18^ Experiments by Hiort, Lincoln and Nordén on [Ru(phen)_2_dppz]^2+^, including the use of dichroism methods, confirmed that both enantiomers intercalated and that the delta not only bound more strongly to B-DNA but was also more emissive than its lambda counterpart.^19^ Further studies with various derivatives of this complex confirmed this effect.^20^ While the ability of the dppz in complexes [Ru(LL)_2_dppz]^2+^ to intercalate between the base-pairs was established in early studies, it was soon realised that there was probably more than one binding mode. This was particularly evident from luminescence lifetime studies, which showed that biexponential analysis was necessary, not only for racemic complexes with natural DNA, but even for individual enantiomers with double-stranded homopolymers such as polydA.polydT or polydG.polydC.^21^ Detailed studies, examining the effect of factors such as loading (*i.e.* [Ru]/[Nucl] ratio), DNA sequence, medium, ionic strength and temperature, have been carried out more recently for [Ru(LL)_2_dppz]^2+^ (LL = phen, bpy) and binuclear complex [μ-c_*x*_(cpdppz)_2_(phen)_2_Ru_2_]^4+^, with the luminescence lifetime data complemented by isothermal calorimetry in some cases.^22^ These show that generally the lifetime is longer for the Δ-enantiomer and for AT-rich DNAs and that the relative amount of the long-lived fraction increased with loading. The authors suggested that these results might be explained by three different binding geometries and they note that ‘Sensitivity to so many parameters make it difficult to interpret photophysical changes, when mixed or unusual sequences or racemic mixtures are used’.

One might expect that NMR methods would allow the determination of the structure of the intercalated assembly. Such studies are carried out with oligodeoxynucleotides (ODNs). Using these small DNA molecules, potentially permits the study of the binding sites in mixed sequence DNA. However, interpretation of the results from such experiments is not straightforward, in part because the resonances at room temperature are usually broad, due to intermediate exchange kinetics. By working at higher or lower temperatures the signals may be sharpened, and this approach has been used for the enantiomers of [Ru(phen-*d*_8_)_2_dppz]^2+^ (ref. 23*a*) or Δ-[Ru(2,9-Me_2_phen)_2_dppz]^2+^ (ref. 23*b*) bound to {d(GTCGAC)}_2_. In the earlier study, working at temperatures near 0 °C, it was demonstrated through the distinct resonance patterns for the 4,7-protons of the dppz ligand that Δ- or Λ-[Ru(phen-*d*_8_)_2_dppz]^2+^ had different orientations in the intercalation pocket, and it was proposed that the complexes entered from the major groove. For Δ-[Ru(2,9-Me_2_phen)_2_dppz]^2+^ evidence for intercalation was inferred from the upfield shifts of the dppz protons and the imino protons of the T_2_ and G_4_ nucleobases. NOESY measurements indicated that the complex entered from the minor groove with the principal binding site involving intercalation at G_4_A_5_ or A_5_C_6_, and it was proposed that a similar binding would also be found with [Ru(phen)_2_dppz]^2+^.^23*b*^ More recently, an NMR study has been reported with the achiral [Ru(tpm)(dppz)py]^2+^ (tpm = tripyridazolemethane; py = pyridine)^24^ bound to d(AGAGCTCT)_2_ and d(CGAGCTCG)_2_, which showed that the dppz ligand intercalates into the G_2_A_3_ step from the minor groove. In this study two differing orientations of the complex in the intercalation cavities were proposed.

Even small changes to the ancillary ligand LL can have a major effect on the DNA binding and the photophysical properties of ruthenium polypyridyl complexes. For example, the group of Glazer has shown that visible light irradiation of complexes such as [Ru(bpy)_2_(6,6′-Me_2_bpy)]^2+^ can lead to the expulsion of the sterically-hindered 6,6′-Me_2_bpy ligand with a strong influence on the photochemical reactions with DNA.^25^ The methyl groups cause distortion of the complex geometry and increase of the Ru–N bond lengths, accounting for the bond weakening.

Another group of ligands which has been studied in detail are ones such as TAP, HAT or 2,2′-bipyrazine where the lowest-lying excited states of complexes containing at least two of the ligands are sufficiently oxidising to extract an electron from guanine. The behaviour of these complexes is discussed in Section 3.2.^26^

It is only in the last five years or so that X-ray structures of dppz complexes bound to small DNA molecules have been reported, and some key features of these are the subject of the next section. Subsequently we describe some of the main aspects of the photophysics and photochemistry of the complexes with an emphasis on recent transient spectroscopy experiments which aim to characterise reactive intermediates. Of special interest is to correlate these photophysical/photochemical properties with the structural insights gained from the X-ray structures. A particularly valuable approach to such a study would be to carry out the transient absorption measurements directly on the crystals, and this is the topic of Section 4.

## 2. Structural studies

The extensive number of structure determinations carried out to determine how [Ru(LL)_2_(dppz)]^2+^ binds with double-stranded DNA has provided very clear evidence that these complexes intercalate into DNA (Table 1). Detailed parameters such as the depth of penetration and orientation of the intercalated dppz ligand and how these factors depend on the nature of the bidentate ancillary ligand LL, enantiomer and the sequence provide a clear rationale for detailed crystallographic study. Structural studies also should provide information about whether the metal complex might change the conformation of the base-pairing nucleotides or modify the overall double helix structure (*e.g.* B-DNA *versus* A-DNA). In this section, some of the correlations which have emerged from these structural results are summarised.

**Table 1:** Reported X-ray structures of [Ru(LL)_2_dppzR_*n*_]^2+^ complexes bound to oligodeoxynucleotides

Complex	Sequence	PDB	Ref.
**Lambda**			
[Ru(TAP)_2_dppz]^2+^	TCGGCGCCGA	3QRN	27
[Ru(TAP)_2_dppz]^2+^	TCGGCGCCGA	4LTG	31
[Ru(TAP)_2_dppz]^2+^	TCGGCGCCIA	4QI0	38
[Ru(TAP)_2_dppz]^2+^	TTGGCGCCAA	5ET2	38
[Ru(TAP)_2_(11-Cl-dppz)]^2+^	TCGGCGCCGA	4III	32
[Ru(TAP)_2_(11-Me-dppz)]^2+^	TCGGCGCCGA	4X18	33
[Ru(TAP)_2_(11-CN-dppz)]^2+^	TCGGCGCCGA	5NBE	35
[Ru(TAP)_2_(10-Me-dppz)]^2+^	TCGGCGCCGA	4MJ9	33
[Ru(TAP)_2_(10,12-Me_2_-dppz)]^2+^	TCGGCGCCGA	4X1A	33
[Ru(TAP)_2_(11,12-Me_2_-dppz)]^2+^	TCGGCGCCGA	4E8S	34
[Ru(TAP)_2_(11,12-F_2_-dppz)]^2+^	TCGGCGCCGA	4MS5	34
[Ru(phen)_2_(dppz)]^2+^	CCGGTACCGG	3U38	29
[Ru(phen)_2_(dppz)]^2+^	CCGGATCCGG	4E7Y	29
[Ru(phen)_2_(dppz)]^2+^	(^Br^C)GGC/GCCG	5LFW	30
[Ru(phen)_2_(11,12-Me_2_dppz)]^2+^	(^Br^C)GGC/GCCG	5LFX	30
[Ru(bpy)_2_(dppz)]^2+^	(^Br^C)GGC/GCCG	5LFS	30

**Delta**			
[Ru(phen)_2_dppz]^2+^	TCGGCGCCGA	5JEU	37
[Ru(phen)_2_dppz]^2+^	TCGGCGCCGA	5JEV	37
[Ru(bpy)_2_dppz]^2+^	CGGAAATTACCG	4E1U	36

**Lambda & delta**			
[Ru(phen)_2_(dppz)]^2+^	ATGCAT	4JD8	28

### 2.1 DNA binding modes of ruthenium dppz complexes – the evidence from X-ray crystallography

The first X-ray structural study of DNA-bound ruthenium dppz complexes, that of [Ru(TAP)_2_dppz]^2+^ bound to d(TCGGCGCCGA)_2_, was reported in 2011,^27^ and since then a series of other structures have been published by the Cardin group and others.^28–37^ Table 1 shows that to date we know more about the binding modes in crystals of lambda complexes. This may be due in part due to the choice of DNA sequences. Crystals containing only the lambda enantiomer form from a racemic mixture with some of the sequences used. In others, crystallisation only, or perhaps more readily, occurs from a solution of one enantiomer of complex. In yet other cases no crystals are obtained. A systematic approach to crystallisation is ongoing in the Cardin group.

**Fig. 2:**
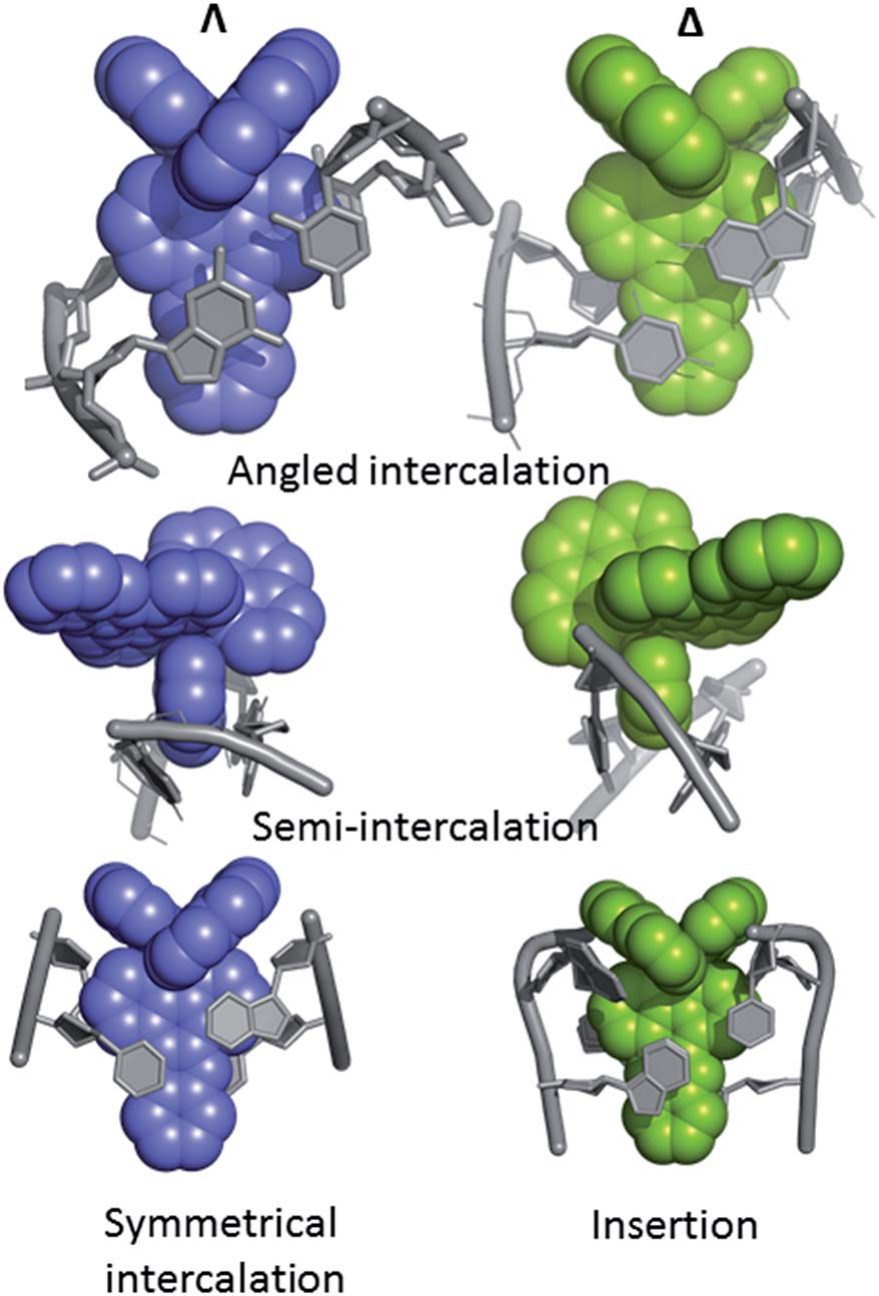
Summary of the main binding modes of lambda (blue) and delta (green) complexes with duplex DNA. The sequence selectivity of these modes, not shown here, is described in Section 2 of the review.

Even with the restrictions noted above, much can be learnt from structural studies. Crystallography has already provided clear evidence of a range of intercalation modes of the dppz ligand, see Fig. 2, and to some extent of the effect of ancillary ligands, enantiomer and DNA sequence dependence in duplex DNA. Factors determining the depth and orientation of the dppz ligand and the effect of dppz substitution at the 10, 11 and 12 positions (R_1_, R_2_ and R_3_ in Fig. 1) have also been identified, although much more work is required in all these areas. As a bonus, a semi-intercalative (kinked) binding mode has been observed for both enantiomers (Fig. 2). This mode presumably aids the self-assembly process of crystallisation by crosslinking duplexes in the structure. As pointed out in the Introduction this mode of binding was proposed for [Ru(phen)_3_]^2+^ with calf thymus DNA^12^ but no crystals of this complex with oligonucleotides have been reported.

**Fig. 3:**
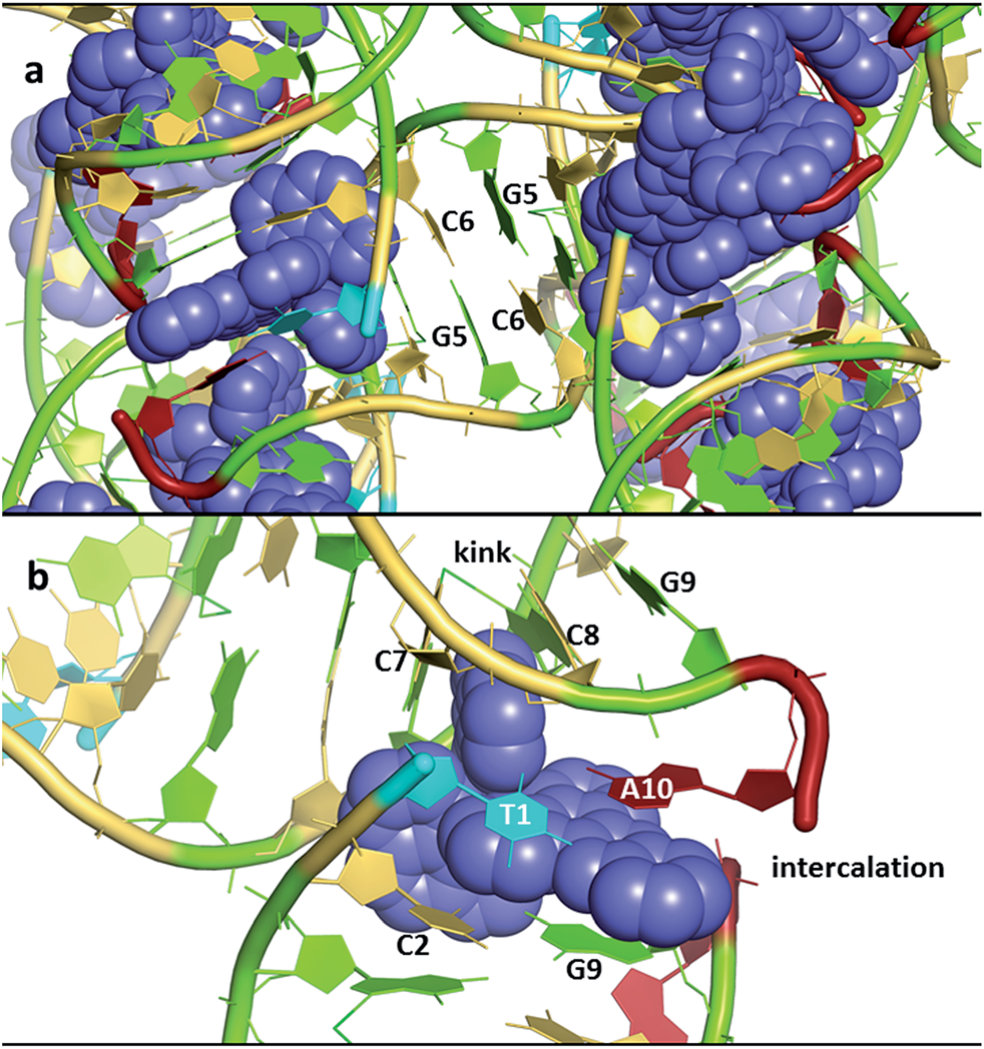
(a) An overview of the structure of the decamer duplex d(TCGGCGCCGA)_2_ with the complex Λ-[Ru(TAP)_2_dppz]^2+^.^27^ The view direction is into the helix twofold axis. PDB code ; 3QRN. (b) The environment of a single complex. The semi-intercalation at basepairs C_7_-G_4_ and C_8_-G_3_ links two duplexes, with the A_10_ of the same strand stacked onto the dppz ligand. The view angle is from the major groove of the duplex which includes T_1_, C_2_ and G_9_. The kinking of a second duplex is marked. The DNA bases are coloured using the Nucleic Acid Database scheme – guanine (green) adenine (red) thymine (cyan) cytosine (yellow).

An overview of the structure^27^ of the decamer duplex d(TCGGCGCCGA)_2_ with the complex Λ-[Ru(TAP)_2_dppz]^2+^ (Fig. 3a) shows another feature of crystal formation – the ruthenium complexes (all equivalent by symmetry in this case) are all separated from one another by at least one layer of DNA, so that there is no direct stacking of one complex on another. There are 18 negative charges per DNA duplex, four of which are neutralised by the Ru cations in this example, so the charge balance is maintained by other cations incorporated from the crystallisation mix. These mixes typically contain a mixture of monovalent and divalent metal cations as well as the tetracationic spermine as polyamine, and the full set of positive charges is typically not identified, even with the exceptional data quality (down to 0.9 Å resolution) available for these studies. Exceptions are the identification of Ba^2+^ and [Co(NH_3_)]_6_^3+^, both standard ingredients of the specialised crystallisation cocktails for nucleic acid crystallography. Fig. 3b shows the environment of one complex, with an intercalation cavity open between G_9_ and A_10_ due to A_10_ base flipping.

**Fig. 4:**
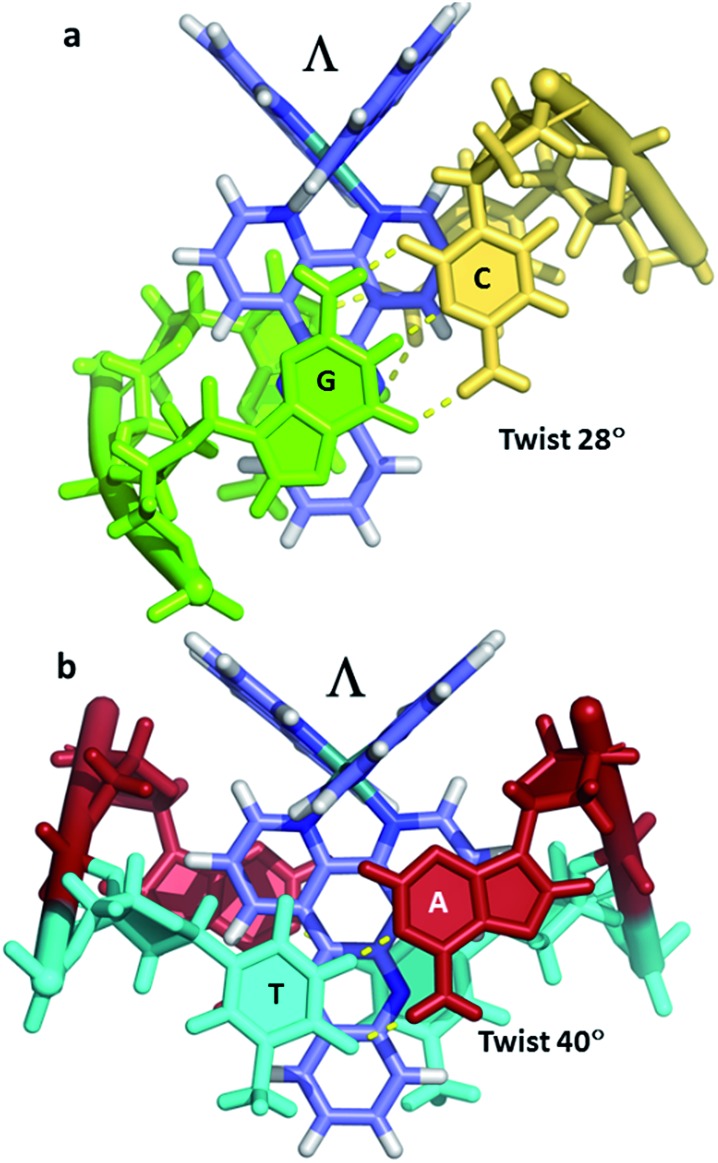
(a) Canted (angled) binding mode at a CC/GG step (4E7Y) (b) symmetrical binding mode at a TA/TA step (; 3U38).^29^

Occasionally the DNA backbone is disordered, but typically there is a remarkable degree of order in the crystals, combined with excellent diffraction properties in many cases, leading to a wealth of detail. The flexibility of the DNA is well illustrated by a remarkable reversible hydration–dehydration, with dehydration inducing extra kink formation.^28^ The overall geometry is best described as a B-DNA, as is typical for intercalation, with a detailed analysis of conformational parameters in general not very revealing. What may be characteristic is the lower twist angle, around 20–30°, associated with the angled intercalation, compared with the higher twist angle (40–45°) associated with symmetrical intercalation, as shown in Fig. 2 and 4.^29^ Some of these features are helpful in interpreting luminescence and electron transfer data as described below.

### 2.2 B-DNA duplexes – intercalation geometries and sequence specificities

There is a general expectation that planar aromatic cations of suitable dimensions to fit a DNA cavity will normally bind by intercalation. In the case of the ruthenium polypyridyl cations containing dppz, all the X-ray evidence is that intercalation occurs from the minor groove side, placing the dppz group between two base pairs and the ruthenium atom between the negatively charged phosphate groups. Beyond this, the precise relationship between a [Ru(LL)_2_dppz]^2+^ complex and DNA cavity is determined by the enantiomer of the complex, the ancillary LL ligand (phen, TAP, bpy) and any dppz substitution. For the DNA, there are 10 possible DNA steps, so our knowledge of sequence dependence is currently incomplete. In addition, an extremely limited range of ancillary ligands have been studied to date (bpy, phen and TAP), as shown in Table 1. There is no structural evidence to date that these ancillary ligands affect the intercalation geometry, although there can be effects on the crystal packing and stability. The depth of intercalation and the chromophore orientation may determine some spectroscopic properties, and there can also be specific stabilising interactions in the minor groove between the ancillary ligands. The extent of the stacking interaction between dppz and the DNA bases of the cavity is determined by the base sequence and the alignment of the dppz long axis relative to the long axes of the base pairs. These are the expected stacking interactions, but the structures suggest that there is also an attractive component in the sugar-ancillary ligand contacts.

#### 2.2.1 The lambda enantiomer

**Fig. 5:**
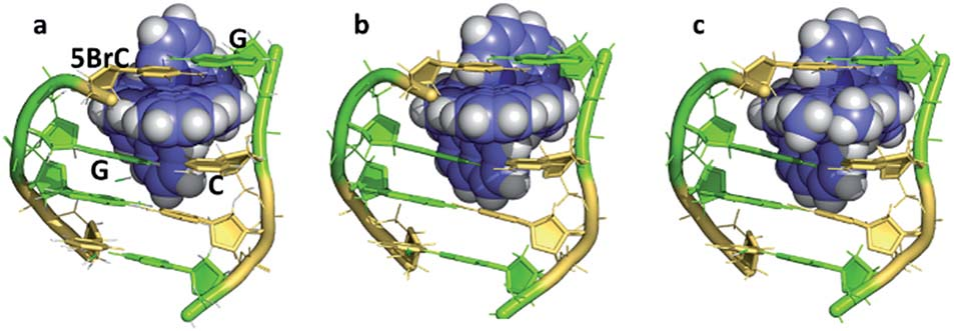
The DNA tetramer d((5BrC)GGC/GCCG) with the complexes (a) Λ-[Ru(bpy)_2_dppz]^2+^ (; 5LFS); (b) Λ-[Ru(phen)_2_dppz]^2+^ (; 5LFW); (c) Λ-[Ru(phen)_2_ 11,12-dimethyldppz]^2+^ (; 5LFX).^30^

**Fig. 6:**
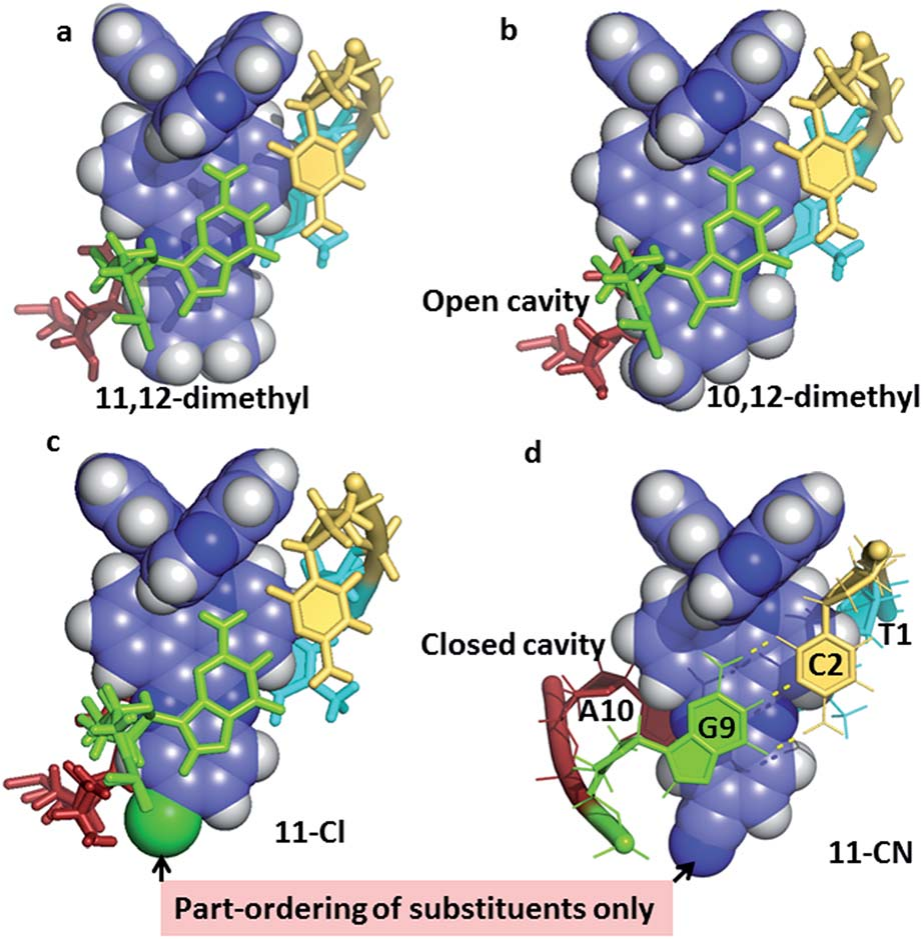
The effect of asymmetric dppz substitution – completely specific orientation of methyl groups at the 10- and 12-positions of dppz. The orientation matches that of the long axis of the GC base pair, with the two methyl groups projecting into the major groove. With the 11-cyano and 11-chloro substituents, the orientation effect is only partial, with the cavity being complete only for the 11-CN. Coordinates from (a) 4E8S;^34^ (b) ; 4X1A;^33^ (c) ; 4III;^32^ (d) to be published.^35^

The lambda enantiomer of [Ru(LL)_2_dppz]^2+^ (LL = bpy, phen, TAP) has been crystallised in more sequences and DNA steps,^27–35^ as well as a wider range of ruthenium complexes, than its delta enantiomer. A representative intercalation cavity is that at a terminal CC/GG step,^29^ (Fig. 4) although binding is not sequence specific, and has also been seen at CG/CG^30^ and TC/AG^27,31^ steps. This canted mode is characterised by a low helical twist, optimising the base stacking onto the dppz chromophore. At an unsymmetrical step such as CC/GG, in principle there are two orientations of the canting, as there are two distinct sides to the intercalation cavity. The preferred orientation is one which maximises the purine/dppz stacking interactions, as shown in Fig. 4a, with further examples in Fig. 5 and 6. All these structures show the same angled orientation of the dppz ligand in the cavity, with one face of the ancillary ligand (TAP, phen or bpy) directly contacting the sugar ring and the consequence that the second ancillary ligand is almost perpendicular to the long axis of the base pairs, exposed in the minor groove of a helical DNA strand. In the case of Λ-[Ru(phen)_2_dppz]^2+^ the symmetrical binding mode is so far specific for the TA/TA step (and not *e.g.* the AT/AT step),^29^ and is characterised by higher twist angle (40°) and the packing of the ancillary phen ligands against, in this case, both, not just one, of the sugars. The high twist suggests a possible hydrophobic interaction in the extensive sugar-phen contacts.^29^ In the crystal structures this mode is distinguished by its occurrence on twofold axes in the crystal lattice, which in the case of symmetrical self-complementary DNA decamer duplexes, coincides with the central unique step of the duplex. This site preference must be primarily due to the increased depth of intercalation possible when the purine is adenine, even though there is limited stacking overlap between the dppz chromophore and the bases. This step has the weakest stacking interaction of the 10 possible base steps, which seems to be the most convincing reason for the binding specificity in this mode. The preferred binding site in the d(CCGGTACCGG)_2_ duplex in solution was subsequently shown to be at this step (see below).^29^

Fig. 5 compares three further examples of this angled binding mode, at the symmetrical CG/CG step, showing that bpy and phen ancillary ligands give isomorphous structures, also with a twist of 28°.^30^ Fig. 5c also shows symmetrical dimethyl substitution of the dppz ligand, at the terminal 11- and 12-positions. Because of the angled intercalation, the two methyl groups become inequivalent with respect to the cavity.

#### 2.2.2 Dppz substitution in the lambda enantiomer

As well as enantiomer specificity, additional specificity can be introduced by modifications to the dppz ligand.^30,32–35^ Prior to the establishment of canted minor groove intercalation as the predominant binding mode, except for the TA/TA preference, a range of symmetrically substituted derivatives had been synthesised. It now seems very likely, given the predominance of angled binding modes, and the clear orientational preferences exhibited to date, that asymmetric substitution should be the subject of further work. To examine these details, it is necessary to say a little more about the framework used for these experiments, designed around a readily reproducible crystallisation,^27^ giving highly diffracting crystals (Table 1), that of the Λ-[Ru(TAP)_2_dppz]^2+^ + d(TCGGCGCCGA)_2_ system (Fig. 3). This crystal assembly is an excellent testbed for the effects of dppz substitution, as the terminal ring projects into the major groove and solvent space within the crystal, therefore allowing for modification and a reasonable degree of confidence that useful comparisons can be made within an isostructural framework. Such comparisons are an added advantage of working with crystals, minimising as it does the number of variables to be considered. A minor apparent disadvantage to this choice of system is that the terminal TA basepair is not closed, instead the adenine is flipped out and stacks and hydrogen bonds with a symmetry related thymine base.

The example in Fig. 6a, of Λ-[Ru(TAP)_2_(11,12-dimethyldppz)]^2+^, shows how the alignment maximises the dppz-guanine stacking, but highlights the resulting asymmetry of the methyl substituents, one of which protrudes much further into the major groove. Fig. 6b shows the corresponding 10,12-derivative with the protruding major groove face of the ligand aligned with the long axis of the GC base pair, and no sign of methyl group disorder in the very high quality resulting diffraction maps.^33^ Fig. 6c and d show the effects of 11-Cl^32^ and 11-CN^35^ substitution. Here the ordering of substituents is only partial, though the major orientation is clear, and is on the purine side of the cavity. Although the cyano substituent does not give a strongly asymmetric binding, it does result in the formation of a closed intercalation cavity, the first example in this system.

#### 2.2.3 The delta enantiomer

**Fig. 7:**
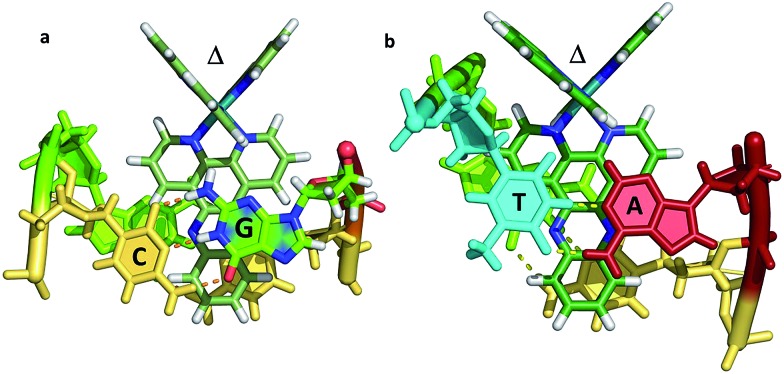
The two known examples of intercalation by delta enantiomers. (a) Δ-[Ru(bpy)_2_dppz]^2+^ at a CG/CG step^37^ (b) Δ-[Ru(phen)_2_dppz]^2+^ at a TG/CA step.^36^ (PDB codes ; 4E1U and ; 4JD8).

The delta enantiomer is represented by only two examples of intercalation,^36,37^ shown in Fig. 7. In both cases the resulting geometry is both sequence dependent and clearly influenced by binding of additional complexes at adjacent steps in the sequences. In the first example there is the steric effect of an adjacent complex at the AA mismatch insertion site (Fig. 7a).^36^ In the second (Fig. 7b) the presence of the lambda enantiomer at the adjacent TG/CA step in the hexamer duplex d(ATGCAT)_2_) unwinds the duplex and could also have a steric effect.^37^ In (a), the effect is to give almost symmetrical intercalation at this CG/CG step whereas, in (b) the complex is acutely angled, contrasting with an almost perpendicular orientation of the adjacent lambda enantiomer, so that neither can be said to be completely independent of the other enantiomer.

It is therefore not so simple to generalise from the two examples currently available as to the precise geometry to be expected in different binding situations. The topic of mismatch recognition is specifically covered below (Section 2.4).

#### 2.2.4 The shape of the minor groove

**Fig. 8:**
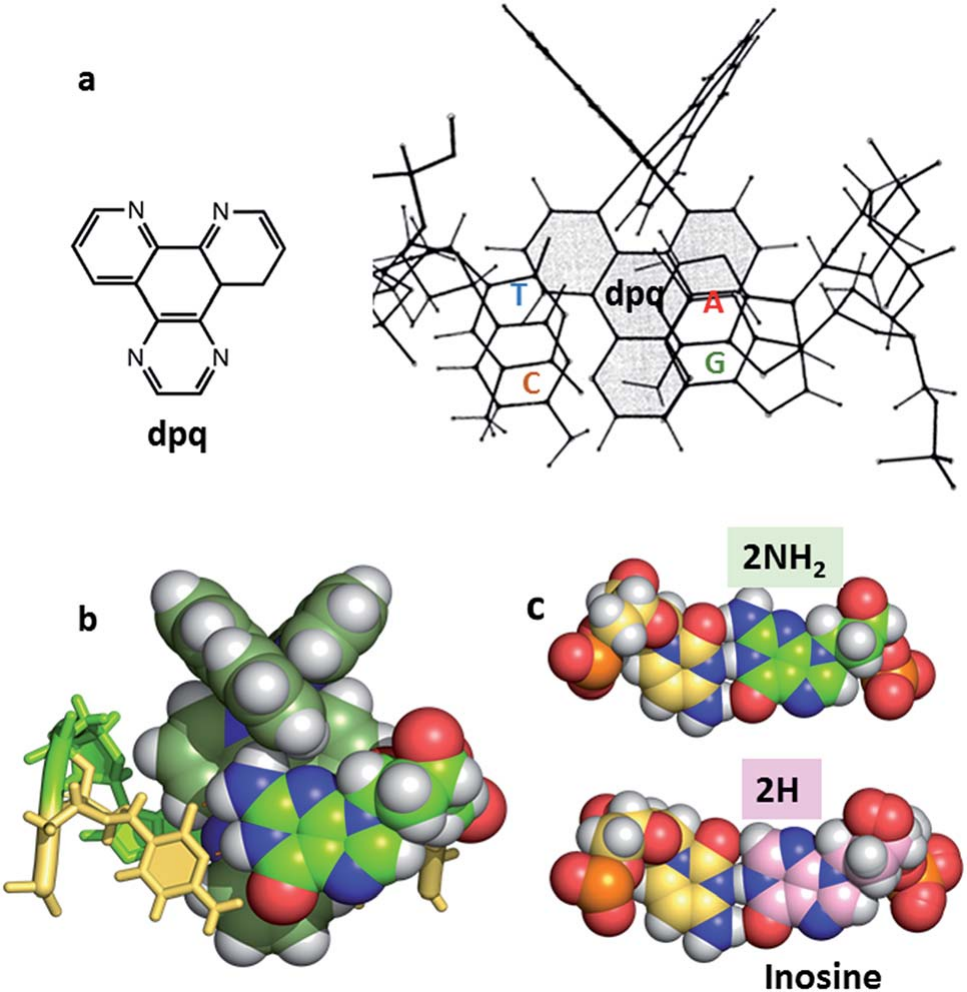
(a) Original NMR model for minor groove intercalation of the dpq ligand in [Ru (phen)_2_dpq]^2+^ (ref. 23*c*) (b) space-filling model showing the effect of the –2NH_2_ of guanine in the minor groove on the depth of intercalation of a Ru–dppz complex.^31^ (; 4JD8) (c) Comparison of the G–C and I–C base pairs with respect to the minor groove.

The known structures listed in Table 1 consistently show the presence of stabilising interactions, which, like the base pairing of DNA, are integral to its structure, and drive the self-assembly of crystal packings. One such constant feature is the effect of guanine on the depth of intercalation, because of the presence of the –2NH_2_ group in the minor groove which is absent in adenine. The effect is shown in Fig. 8b and c, together with an early NMR model of Fig. 8a.^23*c*^

In many cases it is the presence of this extra substituent in the minor groove which determines the depth of intercalation, and can also determine a binding preference for adenine–thymine containing sequences. Typically, this amino group is in contact with an ancillary ligand, in this case bpy. This effect is seen for both enantiomers and over a range of orientations. We have recently argued^30^ that symmetrical intercalation at CG/CG steps is disfavoured by this steric effect in a way not seen at TA/TA steps, leading to a better understanding of why the symmetrical binding at the TA/TA step seems to be unique (see below).^30^

#### 2.2.5 Nonstandard bases

As part of our survey of methods for the study of transient guanine oxidation, the use of the base inosine was explored. As Fig. 8c shows, the steric effect of inosine substitution is, by replacing the 2-NH_2_ group of guanine by 2-H, to generate a minor groove surface structurally identical to that of a TA basepair, although the major grooves would be different. The sequence d(TCGGCGCCIA) crystallised with Λ-[Ru(TAP)_2_dppz]^2+^ to give a highly diffracting structure (Table 1) isomorphous with that obtained with d(TCGGCGCCGA) and a cavity like those shown in Fig. 5a–c.^38^ A structural difference is that the metal complex is more deeply intercalated, by about 0.4 Å. Interestingly, the use of the d(TTGGCGCCAA) sequence, which generates a terminal TT/AA cavity, gives significantly smaller change in intercalation depth of about 0.3 Å.^38^ Therefore, there must also be an effect of the electronic difference between a CI and a TA basepair, and this experiment demonstrates the complexity of apparently simple substitutions and the need for caution in general.

### 2.3 Semi-intercalation (kinking)

**Fig. 9:**
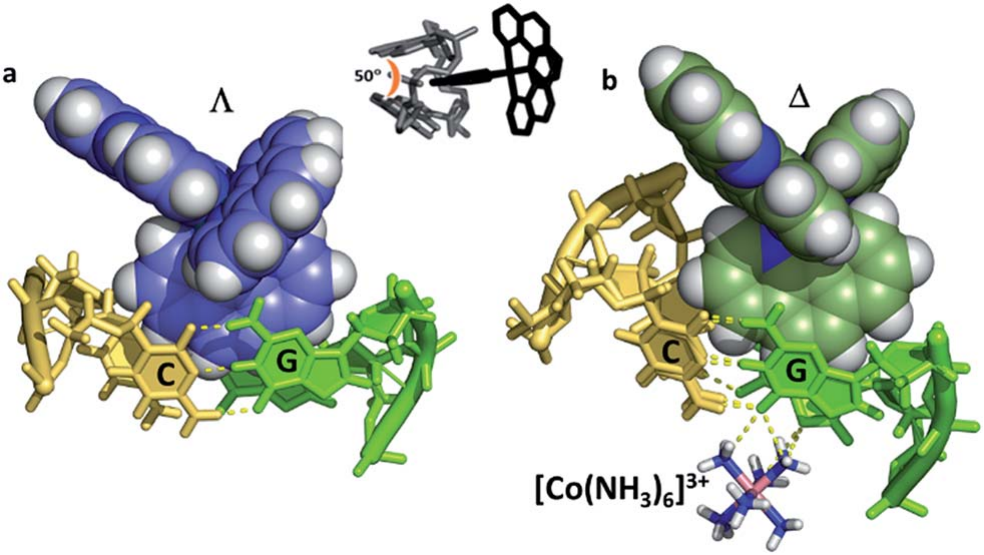
Semi-intercalation (kinking) at CC/GG steps for (a) lambda^29^ (; 4E7Y), and (b) delta^37^ (; 5JEV) enantiomers. There is a very consistent kink angle of 50°.

In our work with both phen and TAP complexes, we have been fortunate in that a side-effect of working with these ancillary ligands has been the ability of both phen and TAP ligands to kink sequences such as d(TCGGCGCCGA)_2_ at the GG/CC steps, as shown in Fig. 9. Because of these ligand choices, we see self-assembly of the whole three dimensional crystal framework with its combination of intercalative and kinked binding modes (Fig. 3b shows this combination linking two duplexes^27^). The resulting structure has a robustness which is rare in biologically related assemblies and hence particularly valuable in the studies described in this review.

This kinked binding mode has an importance of its own because DNA kinking is an integral part of the functioning of many enzymes which act on DNA to repair damage, and the presence of a kink (such as that induced by cisplatin^39^) can induce apoptosis. Kinking by platinum complexes is towards the DNA major groove, widening the minor groove and hence permitting different forms of minor groove recognition.

The kinking by phen^29^ and TAP^27^ ligands (so far not observed for bpy) gives a characteristic binding motif for these complexes, which has a consistent geometry between structures of an approximately 50° sharp bend, and is independent of complex chirality, with examples known for both enantiomers. To date semi-intercalation has only been seen in crystals at CC/GG steps, and has not been the subject of a systematic study. The presence of the kink creates a coordination environment in the major groove which can accommodate cations such as Ba^2+^ and [Co(NH_3_)_6_]^3+^, and although the cations are not an integral part of kink formation, perhaps they assist crystal formation.

### 2.4 Mismatches, insertion and *syn*-adenine flipping with delta complexes

**Fig. 10:**
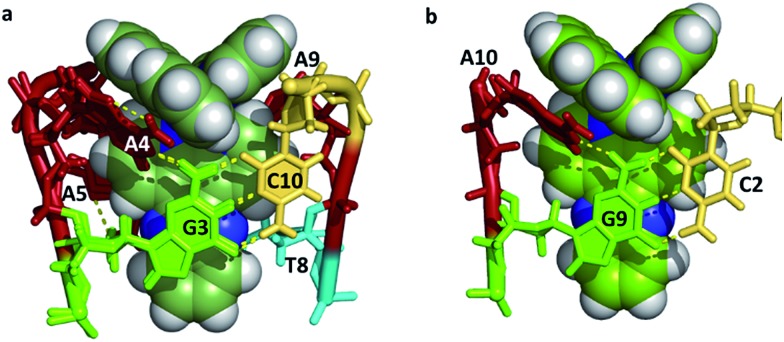
(a) A–A mismatch recognition in the structure of Δ-[Ru(bpy)_2_dppz]^2+^ + d(CGGAAATTACCG)_2_.^32^ (; 4E1U); (b) [Ru(phen)_2_dppz]^2+^ + d(TCGGCGCCGA)_2_ showing end-capping (; 5JEU).^31^

Mismatch recognition could form the basis of a valuable diagnostic tool, and an important aim for designers of new complexes, since mutations in genomic DNA lead to mismatches. Inactivation of mismatch repair pathways is often found in cancerous cells. There are twelve possible mismatches, but so far only one has been structurally characterised^36^ using a modified version of the well-known oligomer sequence d(CGCAAATTTGCG). Clues to the design of specificity for mismatch recognition are provided by an A–A basepair mismatch, in which symmetrical flipping out of the two mismatched adenine bases is possible. This flipping creates a step at which a specific delta enantiomer recognition has been demonstrated using the combination Δ-[Ru(bpy)_2_dppz]^2+^ + d(CGGAAATTACCG). Four complexes bound in total to this sequence, two by intercalation and two at the two A–A mismatches.^36^ The complexes are therefore sandwiched between the adjacent GC and TA basepairs. The intercalative binding has already been illustrated in Fig. 7a and the insertion mode is shown in Fig. 10, seen from the GC side.

In the insertion binding mode, the complex is bound in the minor groove, with the two mismatched adenines flipped out and stacked on the ancillary bpy ligands, with a *syn* conformation of each nucleoside. On the CG side of the cavity, the assembly is stabilised by the formation of an additional hydrogen bond between the guanine 2-NH_2_ group (G_3_ in Fig. 7) and the adenine N_1_ position, in addition to the stacking interaction between the adenine ring and the bpy ligand. This additional hydrogen bond is shown in Fig. 10 linking the canonical GC basepair to the flipped out adenine A_4_, and thus tethering the flipped out base to the main helix.

This tethering is facilitated by the orientation of base A4 stacking onto the aromatic and hydrophobic bpy surface. This assembly therefore combines the stacking of guanine G3 onto a dppz ligand with the additional stacking of adenine A4 onto the bpy ligand and the formation of an A_4_–G_3_ hydrogen bond. This arrangement is only stereochemically allowed for delta complexes, but should also be possible if phen is the ancillary ligand. In a racemic mixture, this binding mode should therefore selectively bind delta complexes.

There is no other structural literature of mismatches, but a search for structures showing the same sort of stacking on ancillary ligands provides two examples, in both cases where the complex has delta stereochemistry, as would seem essential for this binding mode. The binding of Δ-[Ru(phen)_2_dppz]^2+^ to d(TCGGCGCCGA)^37^ (Fig. 10b) has features similar to that of the mismatched structure discussed above, as the AT base-pair flips open and the dppz therefore endcaps by binding to the C_2_G_9_ base-pair. The extruded A and T stack with phen ligands from other complexes. An example of a binuclear complex with a binding mode which includes adenine flipping is the dinuclear complex [μ-(11,11′-bidppz)(phen)_4_Ru_2_]^4+^ bound to d(CGTACG).^40^ In this case a dppz ligand inserts into the DNA stack similarly, with the extrusion of an AT base pair. In both examples there is *syn*-adenine stacked on the ancillary phen ligand with an additional stabilising hydrogen bond to a 5′ guanine either adjacent or one base removed.

### 2.5 The water content of crystals

**Fig. 11:**
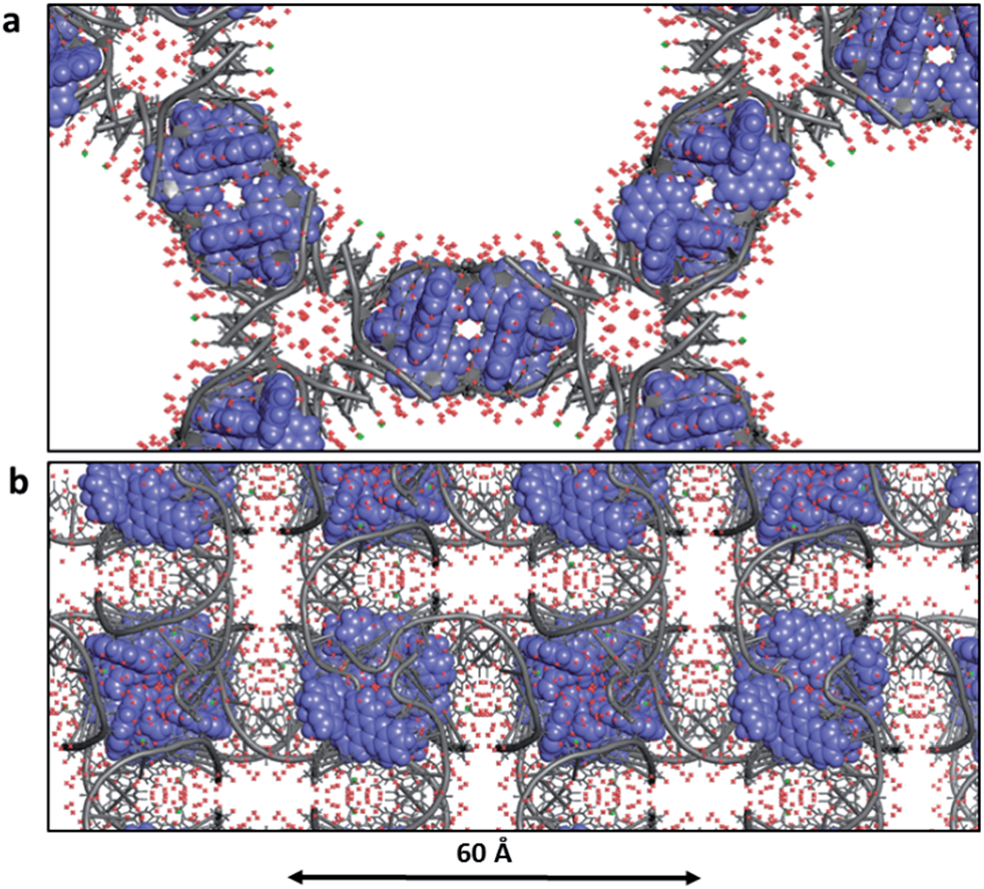
Water in ruthenium-DNA crystals. (a) A structure with a high content of disordered water; Λ-[Ru(phen)_2_dppz]^2+^ bound to d((5BrC)GGC/GCCG) (; 5LFW).^30^ Projection down the *c* axial direction in space group *P*6_4_22. (b) A more typical example, that of Λ-[Ru(TAP)_2_dppz]^2+^ bound to the d(TCGGCGCCGA)_2_ duplex (; 3QRN).^27^ Projection down the *c* axis in space group *P*4_3_2_1_2. Water molecules shown as red crosses in both cases.

Biological crystallographers always work with crystals containing water, and the crystallinity is immediately lost in the absence of water. This point is worth highlighting, as it is so different from the experience of chemical crystallographers. For example, for infrared spectroscopic work with crystals or for neutron diffraction study, the water may be replaced with D_2_O, without loss of fidelity. The typical water content of DNA crystals is 40–60%, but in the case of Λ-[Ru(phen)_2_dppz]^2+^ bound to d((5BrC)GGC/GCCG)^30^ the water content is as high as 72% with solvent channels running right through the crystal lattice (Fig. 11a). In this example, which in this respect is typical, there are typically up to three layers of ordered water molecules before the electron density maps show no clear ordering. The solvent channel here is about 54 Å across. Fig. 11b shows the large amount of ordered water which can be identified in a 1 Å resolution structure – in this case that of Λ-[Ru(TAP)_2_dppz]^2+^ bound to the d(TCGGCGCCGA)_2_ duplex.^27^ This lattice still allows for the free movement of water, and hence the practicality of complete deuterium exchange with all the exchangeable H atoms of the crystal. In this example, there are 74 water molecules located per DNA strand (148 per duplex) of which 58 make direct hydrogen bonds to the DNA (78% of all waters). Adding together both these ordered waters and the volume of the void space gives the total water content as 65%. Thus, water is the major component of the crystal structures here and essential to crystal stability. A controlled dehydration study of the crystal represented in Fig. 3a and 11b (at room temperature) demonstrated the flexibility of the nucleic acid component of these crystals.^31^

## 3. Transient spectroscopy studies of dppz complexes in solution

As discussed in the Introduction, there have been a very significant number of photophysical studies on [Ru(LL)_2_dppz]^2+^, with a particular emphasis on the DNA light-switching properties of the bpy or phen complexes. While steady state luminescence measurements are a very useful method for monitoring the binding to DNA, time-resolved measurements provide much richer information about the influence of the environment on the excited state, especially as most studies in solution reveal multiple emitting species. The other class of compounds which has been thoroughly investigated are those containing two strongly electron accepting ligands such as TAP, HAT or 2,2′-bipyrazine.^26^ These complexes generally luminesce in aqueous solution but the emission is quenched when binding to guanine-containing DNA, presumed to be due to photo-induced electron transfer (PET).

Below we review recent studies on both the light-switching and photo-oxidising complexes, with a focus on the results of transient absorption spectroscopy, monitoring both in the UV/visible (TA spectroscopy) or mid-IR (TRIR). For excited states such studies will provide information complementary to that obtained by time-resolved luminescence measurements, but importantly these methods also give kinetic and structural information for non-luminescent species such as radicals formed by PET. A particular focus of recent studies has been to examine the transient spectroscopy of combinations in solution of [Ru(LL)_2_dppz]^2+^ with small defined sequence DNAs, where the crystal structures have been determined for comparison. Structures built from X-ray data can help to understand how factors such as the orientation of the complex in the intercalation pocket affects the photophysical and photochemical properties.

### 3.1 Light-switching behaviour

Solution-based investigations of the photophysical properties of [Ru(LL)_2_dppz]^2+^ have been the topic of many publications since the initial report by the Sauvage group, where it was reported that the LL=bpy complex was essentially non-emissive in aqueous solution but luminesced strongly in organic solvents.^18^ This study was extended further by Murphy and others, who demonstrated how the luminescent lifetime of the excited state of [Ru(bpy)_2_dppz]^2+^ in organic solvents depended strongly on the presence of –OH groups in the medium.^20*e*^ The first measurement of the excited state lifetime of [Ru(phen)_2_dppz]^2+^ in aqueous media was made by the group of Barbara using picosecond transient absorption spectroscopy with laser excitation at 400 nm.^20*f*^ These measurements showed the presence of two excited states; the first had a lifetime of 3 ps and formed a second which had a lifetime of 250 ps in H_2_O. In D_2_O the lifetime determined was 560 ps, indicating that the deactivation process shows a strong isotope effect presumably because the non-radiative processes depends strongly on the -OH or -OD vibration. A subsequent transient absorption and linear dichroism study of Δ-[Ru(phen)_2_dppz]^2+^ showed an additional very short-lived (700 fs) species in aqueous solution and slower processes with lifetimes of 7 and 37 ps when bound to calf thymus DNA.^41^

Further detailed variable temperature luminescence studies were carried out in nitrile and alcoholic solvents.^42^ These studies emphasised that the ‘bright’ and ‘dark’ states are in dynamic equilibrium with enthalpic and entropic factors determining whether the ‘dark’ and ‘bright’ states are lowest lying. It was also concluded that in the ‘dark’ state both phenazine nitrogens (*i.e.* N9 and N14) of the dppz ligand were coordinated to water.^42*c*^

While luminescence and time-resolved visible spectroscopic methods are excellent ways of monitoring transient species, the spectra tend to be rather broad. By contrast the structured bands in vibrational spectra reveal much functional group information. Resonance Raman spectroscopic methods have been shown to be excellent techniques to probe the excited states of polypyridyl complexes in DNA.^43^ Thus McGarvey and coworkers showed by transient resonance Raman (TR^2^) using 8 ns 355 nm laser pulses that when intercalated into DNA, both [Ru(phen)_2_dppz]^2+^ and [Ru(bpy)_2_dppz]^2+^ exhibit a characteristic new band at 1526 cm^–1^.^43*a*^ This band was subsequently assigned to a normal mode of the ‘phen’ region of the dppz on the basis of similar studies with deuterium-substituted derivatives.^43*c*^ Later picosecond time-resolved resonance Raman methods provided evidence for a very short-lived precursor state that in both aqueous and non-aqueous media led to the ‘bright’ state. In water this latter species converted rapidly (<20 ps) to the ‘dark’ state.^43d,e^

**Fig. 12:**
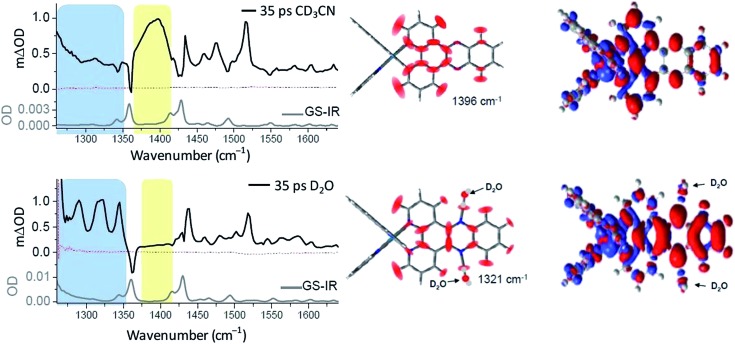
Time-resolved infrared (TRIR) spectra recorded 35 ps after 400 nm excitation of [Ru(phen)_2_dppz]·2PF_6_ (500 μM) in CD_3_CN (top) and [Ru(phen)_2_dppz]·Cl_2_ in D_2_O (right bottom) and their corresponding ground state FTIR (GS-IR) spectra. The blue and yellow coloured regions highlight the characteristic transient bands for the ‘bright’ and ‘dark’ excited states. Graphical illustration of the in-plane vibrational modes of the marker bands of the ‘bright’ and ‘dark’ excited states at 1396 cm^–1^ in CD_3_CN and 1321 cm^–1^ in D_2_O.^45^

The complementary technique of time-resolved infra-red (TRIR) has been increasingly applied to the study transient species.^44^ This method was very recently used to monitor the behaviour of [Ru(phen)_2_dppz]^2+^ in aqueous and non-aqueous solvents and when bound to DNA.^45^ It was shown that the ‘bright’ state in CD_3_CN and the ‘dark’ state in D_2_O have very different spectra in the 1250 to 1600 cm^–1^ region (Fig. 12).^45^ DFT calculations demonstrated that the lowest MLCT excited states were of very different character in CD_3_CN or in D_2_O. Thus the calculated change in electron density distribution between the ground state and the lowest triplet state (Fig. 12) showed an increase on the ‘phen’ portion of the dppz in CD_3_CN, whereas in D_2_O the increase was stronger in the phenazine moiety. (Various approaches to theoretical calculations on DNA-binding dppz complexes bound to DNA have recently been reviewed.^46^) DFT methods were used to predict the infra-red spectra for these ‘bright’ and ‘dark’ states, which were indeed very different from each other and showed the significant spectral features observed experimentally.^45^

**Fig. 13:**
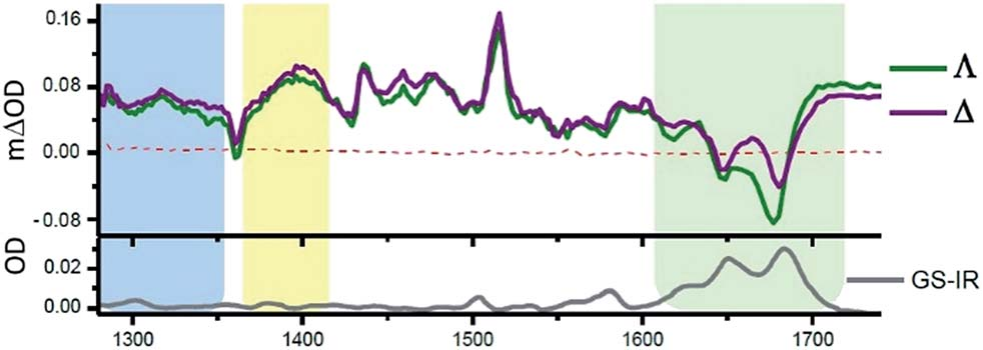
TRIR spectra and recorded 35 ps after 400 nm excitation of Λ-[Ru(phen)_2_dppz]^2+^ (400 μM) and a duplex DNA oligonucleotide d(TCGGCGCCGA)_2_ (500 μM duplex). Bottom panel shows the FTIR of d(TCGGCGCCGA)_2_ (500 μM duplex). All in deuterated potassium phosphate buffer (50 mM) pH 7.^45^

One advantage of the TRIR technique is that for DNA-intercalated species it readily permits one to simultaneously monitor the vibrations of both the transient species produced from the metal complex and the nucleic acid in the immediate environment of the bound species. This can be very powerful as it allows correlation of information of the binding site and the corresponding kinetics. Fig. 13 presents the TRIR of spectra of the Λ- and Δ-enantiomers of [Ru(phen)_2_dppz]^2+^ bound to the DNA duplex d(TCGGCGCCGA)_2_.^45^ It may be observed that in the region below 1600 cm^–1^ the spectra of the two DNA-bound enantiomers are rather similar and have the same spectral features as those found in CD_3_CN, confirming that the species in DNA is closely similar to the ‘bright’ state in organic solvents. By contrast the TRIR spectra in the region between 1600 and 1750 cm^–1^ are strikingly different for the two enantiomers. It is in this area that DNA absorbs strongly, primarily due to the absorptions caused by the carbonyl- and ring-based vibrations, and it may be seen that the strongest bleaching of the absorptions recorded are for those primarily due to the cytosine (1648 cm^–1^) and guanine (1680 cm^–1^). It should be noted that, as the DNA is not directly excited by the 400 nm excitation pulse and as no chemical reaction is expected on this sub-nanosecond timescale, these signals must be caused by a perturbation of the DNA-nucleobases by the proximal excited state. The differing response to the excitation of the two enantiomers must somehow be a consequence of the geometry of their intercalation sites.

**Fig. 14:**
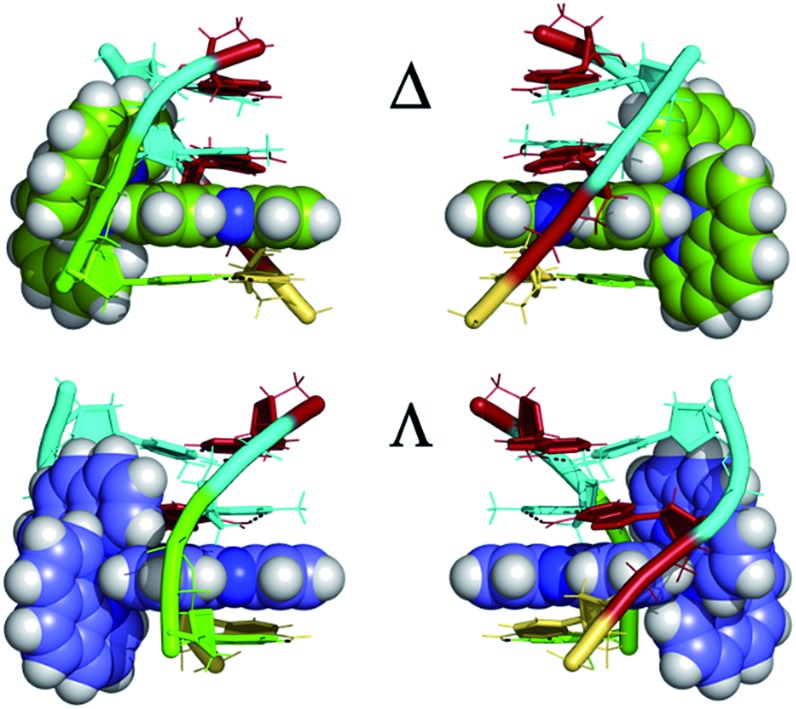
The enantiomer environments in the d(ATGCAT)_2_ structure^28^ PDB code ; 4JD8. For both enantiomers the cavity is asymmetric, with one side in each case having a *trans* conformation for the γ torsion angle and hence exposing the dppz ligand to solvent.

An interesting example where crystal structures may help elucidate the reasons for the differing excited state lifetimes of the enantiomers of [Ru(phen)_2_dppz]^2+^ is shown in Fig. 14. Here both enantiomers are bound to an identical base pair step (TG/CA in the hexamer d(ATGCAT)_2_.^31^ It is the only case in which both enantiomers are bound to the same duplex, and allows us to compare the geometries at the intercalation sites. The different angles of intercalation (87° for Λ and 65° for Δ) results in different exposure of the phenazine nitrogen atoms (*i.e.* N9 and N14) of the dppz ligand to the water molecules, with the Δ complex being more shielded by the sugar phosphate backbone as shown in Fig. 14, which could provide an explanation for its experimentally determined longer lifetime when DNA-bound.

**Fig. 15:**
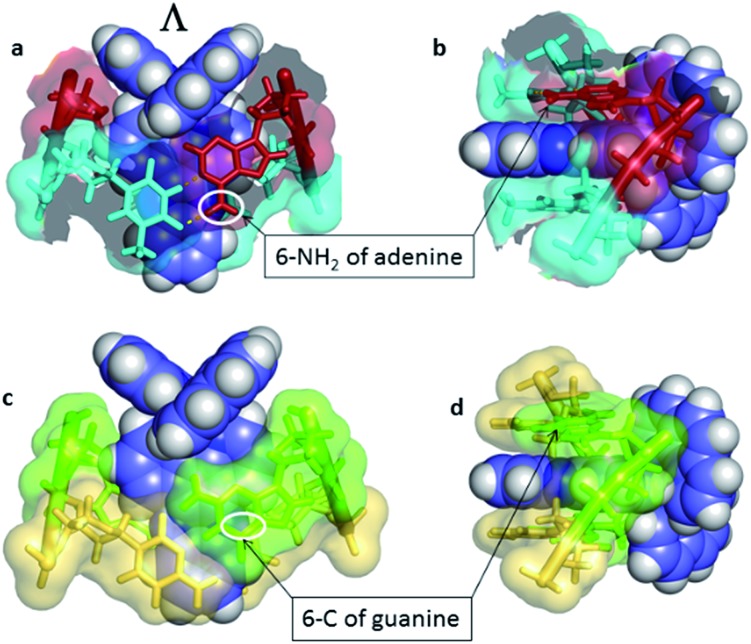
Symmetrical intercalation and the origin of luminescence lifetimes with the lambda enantiomer. (a) and (b) Two views^29^ of a symmetrical TA/TA site showing surface at 50% transparency (PDB code ; 3U38). (c) and (d) A model showing the effect of the 2-NH_2_ group of guanine on the depth of intercalation, derived from PDB entry ; 3U38, drawn in the same style for comparison.^29^ The 6-C of guanine is close to the position occupied by the 6-N of adenine, highlighted by white ovals.

Another relevant crystallographic study is one which reports that the lambda enantiomer binds in a CG/CG site with the characteristic angled orientation.^30^ Symmetrical intercalation has so far only been seen at TA/TA steps (Fig. 4b),^29^ where the exposure of the phenazine nitrogen atoms to water should lower the emission lifetime. By contrast in the corresponding, and so far not structurally characterised, symmetrical intercalation mode at CG/CG, the depth of intercalation would be reduced by ∼1.5 Å by the presence of the 2-NH_2_ group of the guanine base (Fig. 15). The intriguing implication is that the excited state lifetime should not be significantly reduced by water quenching, as the phenazine nitrogen atoms would not be exposed, although experimentally it is found that the lifetime is rather short. It is possible, therefore, that the excited state in GC-containing DNAs may be deactivated by a relatively slow PET as has been proposed earlier.^22^

**Fig. 16:**
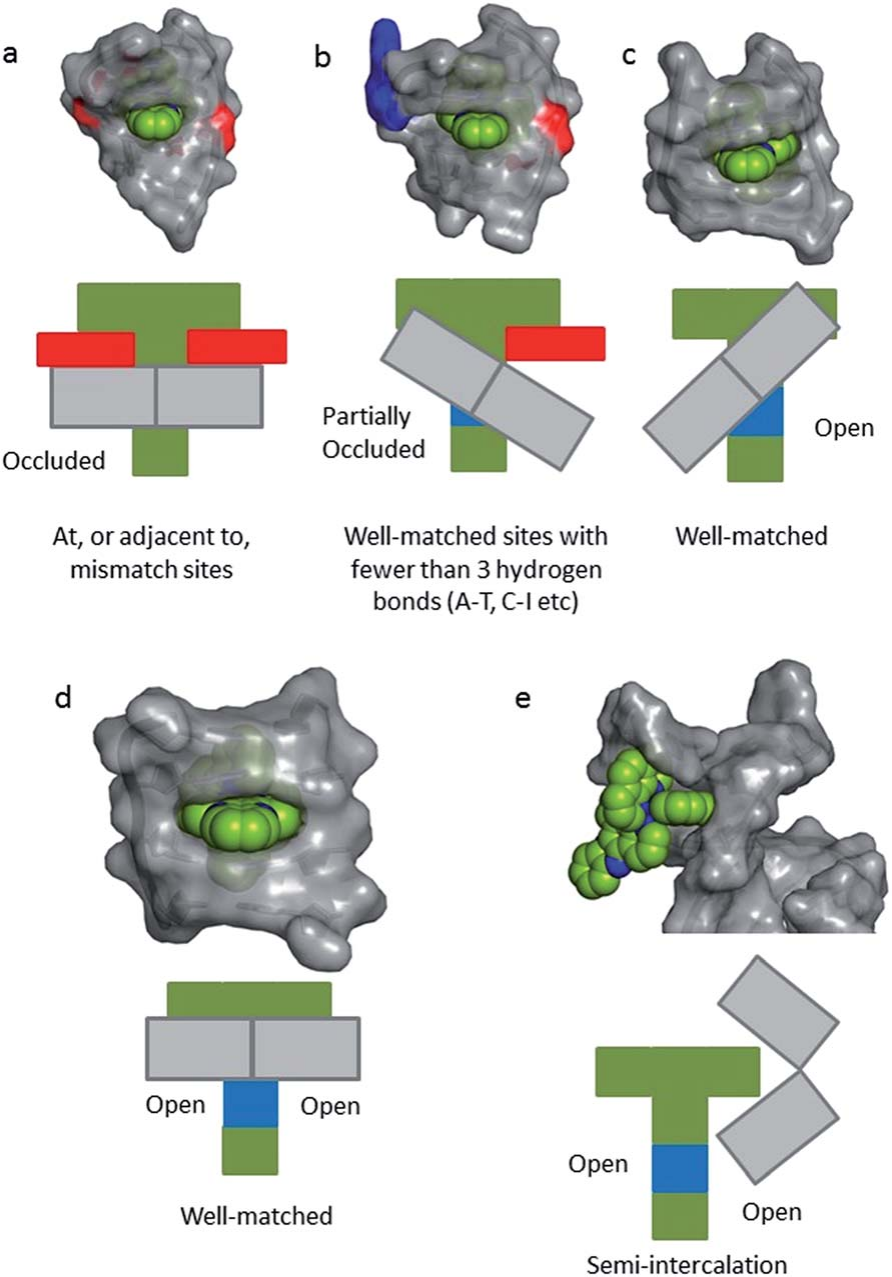
Schematic diagrams for five possible binding modes for Δ-[Ru(phen)_2_(dppz)]^2+^ (green with N9 and N14 atoms of dppz in blue) to DNA (grey and represented by grey blocks with flanking adenine bases as red rectangles). (a) Binding at, or adjacent to, a mismatch site. The flanking purine bases stack on phen, reducing intercalation depth. (b) Insertion into well-matched sites with less than three H-bonds between the bases. The purine may flip out and π-stack onto phen. The pyrimidine may also flip out but does not stack. (c) Canted (angled) intercalation into a well-matched base pair. (d) Symmetrical intercalation at a 5′-AT/AT-3′ step (generated from PDB ; 3U38). (e) Semi-intercalation by phen. In (a) the N9 and N14 atoms in dppz are completely occluded; in (b) one is partially exposed; in (c) one is completely exposed; in(d) and (e) both are exposed.^37^

In a recent paper by Hall *et al.* reporting the crystal structure of the delta enantiomer of [Ru(phen)_2_dppz]^2+^ with the d(TCGGCGCCGA)_2_ duplex, the effect of various binding modes on the emission lifetime was proposed (Fig. 16).^37^ For this enantiomer, considering the access to water, the luminescence lifetime is predicted to be in the following order; mismatch (AA) > well-matched, non-CG site with base flipping ≥ canted intercalation > symmetrical intercalation > semi-intercalation.

### 3.2 Photo-chemical reactions

Another major application of ruthenium polypyridyl complexes has been as photosensitisers. The triplet MLCT excited states can be strong oxidising and/or reducing agents and the long lifetimes means that they can be very effective sensitisers for singlet oxygen.^47^ A major target for such processes are nucleic acids and it was shown in early studies that visible light irradiation of [Ru(phen)_3_]^2+^ or [Ru(bpy)_3_]^2+^ could readily cause single-strand breaks in plasmid closed circular DNA.^3,48^ This process is very easily followed by gel electrophoresis as the electrophoretic mobility of the ‘nicked’ plasmid is much less than the intact form. However, the quantum yield of this process is very low (<10^–5^),^49^ as the assay detects when just one of the more than 7000 phospho-glycosidic bonds is broken.

A more abundant reaction is oxidative damage, which is readily revealed using ^32^P-labelled DNAs and treatment with base after the irradiation.^50^ Such oxidative damage occurs at guanine and in the case of [Ru(phen)_3_]^2+^ with a 2.7 kilobase restriction fragment it was shown that there is a marked variation in the cleavage efficiency, with CGA, TGA, CGT triplets being particularly favoured.^51^ While it is probable that singlet oxygen will play a role in such reactions, it is interesting to note that the reaction can be quite specifically targeted to a site near the photosensitiser, as was shown using oligonucleotide-5′-linked-[Ru(phen)_3_]^2+^ derivative.^52^ This is consistent with the photooxidative damage being induced by a reaction at the nucleobase close to the photosensitiser rather than by a diffusible species such as singlet oxygen. Such a reactive species could be the ruthenium(iii) complex, as these oxidised metallo complexes are known to oxidise guanine.^53^

Turro and coworkers have studied the relative efficiency of cleavage of plasmid DNA using [Ru(bpy)_3_]^2+^, Ru(bpy)_2_dppz]^2+^ or -[Ru(bpy)_2_dppn]^2+^.^54^ They showed that photolysis (455 nm) with [Ru(bpy)_2_dppn]^2+^ led to complete removal of the supercoiled form of the plasmid within 30 s, whereas no change in the DNA occurred for either [Ru(bpy)_3_]^2+^ or [Ru(bpy)_2_dppz]^2+^. The extended hetero-aromatic ligand in [Ru(bpy)_2_dppn]^2+^ causes the lowest lying excited state to be intraligand (ππ*) in character, which is an excellent sensitiser for singlet oxygen. The authors propose that a highly reactive ^3^MLCT state, which can oxidise guanine, is also responsible for the photocleavage.

**Fig. 17:**
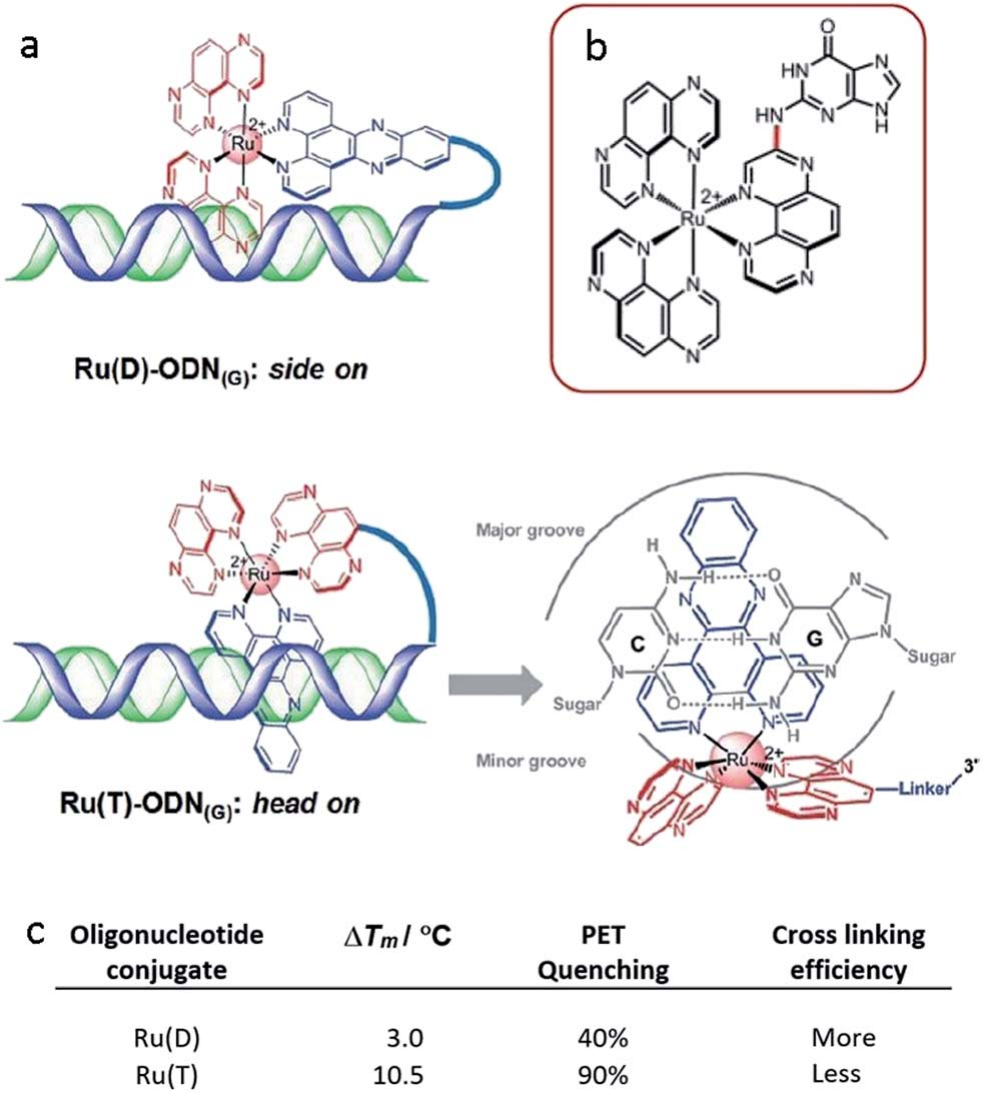
(a) Proposed binding geometries for tethered Ru(D) and Ru(T) complexes, and illustration of the close proximity of the metal centre to a guanine base.^61^ The summary of the measured photo dynamics. (b) Structure of the isolated photo-adduct produced upon illumination of [Ru(TAP)_3_]^2+^ in the presence of a guanine residue.

It is well known that the electrochemical properties of ruthenium polypyridyl complexes can be modified by change of ligands. One system that has been extensively studied in this regard is TAP, where the electron withdrawing nature of the ligand makes the complex a much stronger oxidising agent.^26^ A consequence of this is, for example, that [Ru(TAP)_3_]^2+^ causes significantly more photo-damage to DNA than does [Ru(phen)_3_]^2+^, as was first demonstrated using plasmid DNA to monitor single strand breaks.^55^ However, later work using ^32^P-labelled DNA showed that a more important reaction is the formation of photo-adducts.^51,56^ Subsequently this adduct was isolated and characterised and shown to involve covalent attachment of the TAP ligand to the exocyclic 2-N of guanine (Fig. 17).^57*a*^ Similar behaviour is found for HAT complexes, although in this case the adduct is formed by covalent addition to the 6-O of the guanine.^57*b*^ This behaviour has been exploited extensively by the group of Kirsch-De Mesmaeker to induce covalent cross-linking between a tethered metal complex and another DNA strand, which can have important biological consequences.^58^

**Scheme 1:**
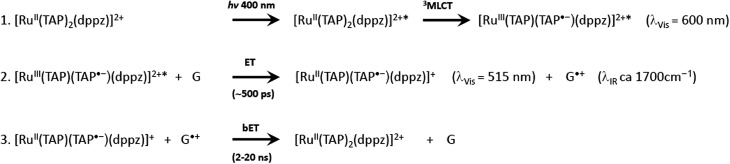
Reaction mechanism showing the photosensitised oxidation of guanine by [Ru(TAP)_2_dppz]^2+^.

It is clear from these early studies that TAP-complexes such as [Ru(TAP)_3_]^2+^ make an interesting system to study the photo-oxidation of DNA, but they suffer from the disadvantage that the binding to DNA is relatively weak and the mode of binding is uncertain (although most probably by semi-intercalation as shown for [Ru(phen)_3_]^2+^).^12^ A much better option is to study complexes which can be expected to bind *via* intercalation. Such a complex is [Ru(TAP)_2_dppz]^2+^, which was found to bind strongly to a wide range of DNAs.^59^ However, unlike its phen analogue, [Ru(TAP)_2_dppz]^2+^ is luminescent in aqueous solution. This emission is quenched when the complex bind to guanine-containing DNA, presumed to be due to electron transfer from guanine to the excited state (Scheme 1), as is the case for other complexes having at least two TAP or HAT ligands.^60^ It is hypothesised that the photoadducts are formed by reaction the transient products of this PET process.

A subsequent detailed study was performed using oligonucleotide conjugates containing a [Ru(TAP)_2_dppz]^2+^ complex attached *via* either (a) the TAP ligand or (b) the dppz ligand, see Fig. 17a.^61^ This study revealed the importance of flexibility on the photoadduct yield. While the intercalation of the dppz resulted in a greater PET, the photoadduct (Fig. 17b and c) yield was observed to be greater under conditions where the TAP ligand was partially intercalated and this was attributed to the greater flexibility of this binding mode afforded by the weaker binding interaction.

#### 3.2.1 Transient spectroscopic studies of [Ru(TAP)_2_X]^2+^ and nucleic acids

To further investigate the primary processes of these photo-oxidation reactions, transient absorption studies were carried out using nanosecond laser flash photolysis. Initial experiments were performed with nucleotides. These showed that the excited state of [Ru(TAP)_3_]^2+^ was efficiently quenched by 5′-guanosine-monophosphate (GMP) at rates close to diffusion controlled but much less efficiently by 5′-adenosine-monophosphate (AMP), and not measurably by the pyrimidine nucleotides.^62^ In the case of GMP, quenching caused the production of the reduced species [Ru(TAP)_2_(TAP˙^–^)]^+^ or [Ru(TAP)_2_(TAPH˙)]^2+^ (p*K*_a_ = 7.6) and the guanine radical (which is formed by deprotonation of the guanine radical cation at neutral pH as it has a p*K*_a_ of 3.9). The back reaction of the reduced species and the guanine radical proceeds with a rate constant close to that of diffusion control. It was also shown that the oxidation of the reduced Ru complex by molecular oxygen proceeded rapidly at pH 9 (2.2 × 10^8^ dm^3^ mol^–1^ s^–1^), but not at pH 6, showing that [Ru(TAP)_2_(TAPH˙)]^2+^ reacts much more slowly with O_2_ than does [Ru(TAP)_2_(TAP˙^–^)]^+^.

As was mentioned earlier, [Ru(TAP)_2_dppz]^2+^ has been shown to intercalate into DNA in solution.^59^ Subsequently a detailed transient spectroscopic study was carried out with the homopolymer {poly(dGdC)}_2_.^63^ Two ultrafast methods were used to probe the spectra and kinetics of transient species living longer than a few picoseconds after excitation with a 400 nm 150 fs laser pulse. Transient absorption (TA) allows the monitoring of spectra in the near UV and visible regions and is particularly useful for monitoring metal complex transients, while time-resolved infra-red (TRIR) is an excellent technique for following reactions of the nucleic acid, as the nucleobases absorb strongly in the region 1500–1750 cm^–1^. Combining these two techniques, it was shown that when bound to polydG.polydC the excited state of the metal complex was reduced with a rate constant of *ca.* (1/500 ps) Probing in the infra-red revealed the reaction of guanine (by ‘bleaching’ of its characteristic absorption at 1690 cm^–1^). This experiment had to be carried out in D_2_O, as H_2_O absorbs strongly in this spectroscopic window. Weak absorption was also noted at *ca.* 1700 cm^–1^, where earlier TRIR experiments had reported that the guanine radical cation absorbed.^64^ The lifetime for the guanine oxidation was determined as *ca.* 700 ps. Further TA experiments confirmed that indeed the reduction of the ruthenium complex was also slower when the reaction was monitored in the deuterated medium, indicating that there is a modest isotope effect. The reverse reaction was too slow to be complete in the time domain monitored by ps-TA, but back reaction lifetimes of *ca.* 9 ns in H_2_O and of *ca.* 14 ns in D_2_O were estimated.

These earlier transient studies of photo-oxidation with [Ru(TAP)_2_dppz]^2+^ and {poly(dGdC)}_2_ were carried out using racemic compounds.^63^ Given the differing features of intercalation (such as site preference, orientation, depth of penetration) exhibited by the enantiomers in oligonucleotide crystals, it was therefore of interest to determine whether these factors would affect the yield and rates of the electron transfer processes. By working in solution with oligonucleotides (and where possible with those where there was crystallographic information) it should be possible to identify whether there are distinct effects of sequence.

**Fig. 18:**
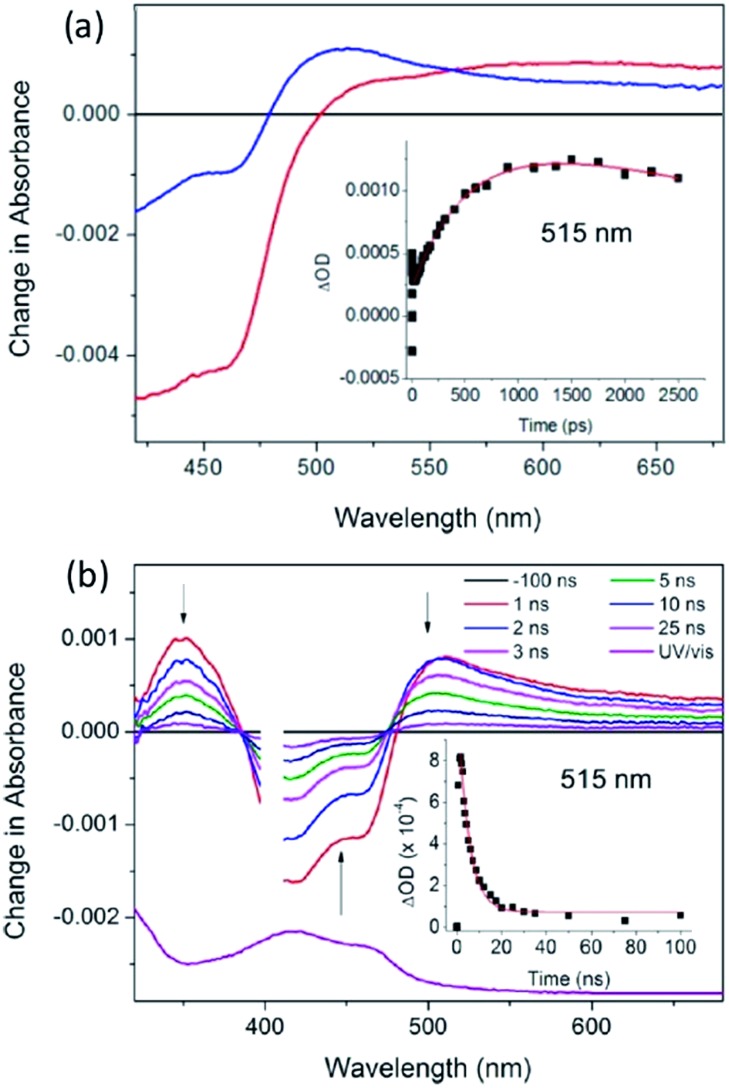
(a) ps-TA spectra (20 ps (red), 2500 ps (blue)) and (b) ns-TA for Λ-[Ru(TAP)_2_(dppz)]^2+^ bound to (G_5_C_5_)_2_, *λ*_exc_ = 400 nm, 1 μJ. Associated kinetic fits at 515 nm (inset). [Ru] = 400 μM, [ODN] = 500 μM duplex in 50 mM phosphate (pH 7) in D_2_O with 50 μm path length.^65^

The first system to be studied was that of [Ru(TAP)_2_dppz]^2+^ bound to the guanine-rich sequence d(TCGGCGCCGA)_2_, for which the crystal structure had been determined.^27^ This oligonucleotide has several guanine environments (*i.e.* CGG, GGC, CGC, CGA) and it was therefore also of interest to compare the results with simpler oligonucleotides containing more uniform environments such as either alternating GC (*e.g.* d(GC)_5_) or blocks of guanines and cytosine (*e.g.* d(G_5_C_5_)). The TA method was used to monitor the yield and kinetics of the photo-oxidation process, exploiting the fact that the excited state has a broad maximum at *ca.* 600 nm while the reduced species peaks at *ca.* 515 nm in the difference spectrum, Fig. 18a.^65^ These measurements revealed that for the Δ enantiomer the yield and kinetics of the photo-oxidation process was similar for each of the three oligonucleotides whereas they varied substantially for the Λ-isomer. The subsequent reaction of the reduced ruthenium complex and the oxidised guanine was readily monitored by nanosecond transient absorption, Fig. 18b. The rate constants for this back reaction were similar (*ca.* 8 ns) for the delta complex bound to any of the three oligonucleotides, but varied substantially for the lambda (5.5 ns for d(G_5_C_5_)); 12 ns for d(GC)_5_ and 17 ns for d(TCGGCGCCGA)_2_. It was proposed that for the Δ-enantiomer this might be a consequence of preferred binding at a GC/GC step which is a common feature of each of the three decamer duplexes. By contrast for the Λ-enantiomer it was suggested that binding at such symmetric steps might be disfavoured as the overlap with the base-pairs is significantly less than for the Δ-isomer and that instead the binding could occur at a GG/CC step. In the case of d(G_5_C_5_) this could also place the photo-oxidising excited state complex at the 5′-side of GG stack which is known to be a hot-spot for oxidation.^66^

**Fig. 19:**
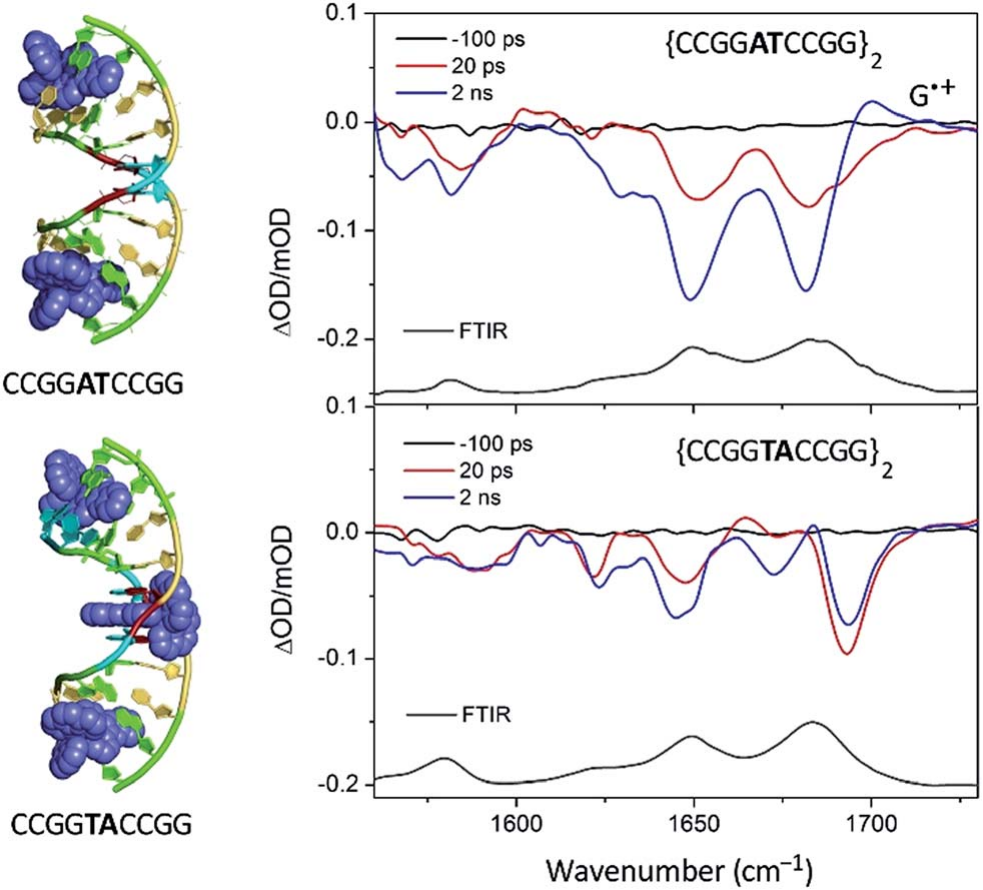
TRIR spectra of Λ-[Ru(TAP)_2_(dppz)]^2+^ bound to d(CCGGTACCGG)_2_ and d(CCGGATCCGG)_2_ recorded at 20 ps and at 2 ns after 400 nm excitation.^67^

As pointed out in Section 2.2.1, the crystal structure obtained with Λ-[Ru(phen)_2_dppz]^2+^ bound to d(CCGGTACCGG)_2_ is, so far, unique in this family of ODNs in that as well as the terminal intercalated complex, a further one is located at the TA/TA step.^29^ This suggests that there is a strong preference for binding at this base-pair step. Assuming that this behaviour would also be found for the almost isostructural Λ-[Ru(TAP)_2_dppz]^2+^, it was anticipated that in solution at a low Ru : Nucl ratio the complex would bind at this step, which is also the only one not containing a guanine. The transient measurements indeed confirm that the yield of electron transfer is very small and that the excited state is relatively long-lived (120 ns) compared to what is observed when the complex is intercalated at a guanine-containing step (10–20 ns).^67^ The TRIR signal shows features consistent with the vibrations of thymine and adenine groups and quite unlike those where the excited state is produced close to a G–C base-pair (Fig. 19). By contrast for Λ-[Ru(TAP)_2_dppz]^2+^ bound to d(CCGGATCCGG)_2_ the yield of guanine oxidation is comparable to that found for d(TCGGCGCCGA)_2_.

The above is an example of where crystallography is an excellent guide to a preferred binding site (and hence to determining control of the yield of PET). However, this is not always the case. For example, as discussed above (Section 2.2.5) substitution of guanine by inosine causes minimal changes to the overall structure of B-DNA, although it does modify the environment of the minor groove. Importantly, as the oxidation potential of inosine is some 200 mV greater than that of guanine,^68^ it is expected that the yield of sensitised photo-oxidation might be significantly reduced. This supposition was tested by comparing the behaviour of [Ru(TAP)_2_dppz]^2+^ in the presence of ODNs where the (i) G_3_, (ii) G_4_, (iii) both G_3_ and G_4_, or (iv) G_6_ in d(TCGGCGCCGA)_2_ had been replaced with inosine. In all these cases the yield of electron transfer was indeed reduced by the I-for-G substitution, although the effect was particularly marked for the Λ-enantiomer.^69^

By contrast when inosine substitution at G_9_ was considered, quite different behaviour was found, as it was observed that the yield and the rates of both forward and back electron transfer were substantially increased for the d(TCGGCGCCIA)_2_ compared to the parent ODN.^38^ This was unexpected as the crystal structures had been found to be isomorphous,^38^ in both cases revealing that the complex bound at the terminal base-pair steps (*i.e.* T_1_C_2_/I_9_A_10_ or T_1_C_2_/G_9_A_10_). This must therefore be attributed to a change in the binding site in solution of the inosine-containing ODN, such that the complex now locates itself at the C_2_G_3_/C_8_I_9_ step and hence is at the 5′-side of the readily oxidisable GG step. The favouring of this step is consistent with entry of the complex from the minor groove (with binding to the inosine-containing site being stronger than to the guanine-containing one, because of the lack of steric hindrance from the 2-NH_2_ group). This example therefore emphasises that caution should be exercised when applying observations in the crystal to determining the binding site in solution.

## 4. Transient spectroscopy studies of dppz complexes in crystals

Understanding the relationship between the geometry of the ruthenium polypyridyl binding site and the ensuing photophysical processes is a significant challenge that has motivated much of the research described in this review. The use of model oligodeoxynucleotides of known base composition and base steps has significantly advanced our understanding. Solution behaviour that is in good agreement with the X-ray structures has been found.^67^ On the other hand, properties that contrast with those predicted by crystallography have been recorded, which raises uncertainty regarding the relevance of a single crystal structure to the often complex solution phenomena.^38^ This gap in understanding can be bridged by performing transient experiments on crystalline systems whose structure has been resolved.^70^

TRIR studies of the photo-dynamics of crystalline samples of [Re(CO)_3_(bpy)Cl] drop cast from saturated organic solutions have previously been reported, which identified some of the challenges working with solid-state forms of photochemically active systems.^71^ Carrying out transient absorption experiments in crystals presents some additional challenges to those normally encountered in solution studies. Firstly, it is important to ensure transmission of the probe beam through the sample. For ruthenium polypyridyl samples this is a limiting factor for UV or visible TA studies, as extinction coefficients tend to be high. By contrast, in the mid-IR, absorbances are much lower, so that TRIR should be a viable technique, if the crystals used have a thickness of no more than a few microns. TRIR of micron-sized crystals also removes the scattering that would be observed in the corresponding UV and visible experiment. Another obstacle to overcome is to ensure that the integrity of the crystals is maintained during the experiment and that the sampling conditions are optimised to ensure that there has been no damage caused by the pump laser beam.

**Fig. 20:**
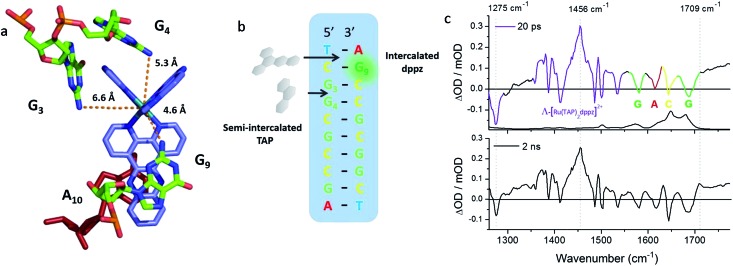
(a) Crystal structure of electron transfer site (PDB:3QRN) showing distances between the Ru atom and the three guanine 2-amino nitrogen atoms in the structure (b) site of oxidation and (c) TRIR spectra after 20 ps and 2 ns following 400 nm (150 fs, 1 μJ, 1 kHz) excitation.^70^

These requirements are all satisfactorily met for the PET study of crystals of Λ-[Ru(TAP)_2_(dppz)]^2+^ bound to (TCGGCGCCGA)_2_, which were chosen as a first example due to their robust nature.^27,28,70^ The packing arrangement in the crystal structure reveals the complex binds across two duplex sequences placing it in the environment of two guanine-containing sites: (i) at the dppz intercalation site at the terminal T_1_C_2_:G_9_A_10_ step the G9 base, which is stacked onto the central pyrazine ring of the dppz. (ii) At the semi-intercalated site a TAP ligand, which is wedged into the G_3_G_4_:C_7_C_8_ step of the other sequence (Fig. 20a and b). This presents three possible sites of guanine oxidation. The challenge is to identify the more likely target.

The TRIR spectra recorded after 400 nm excitation of D_2_O exchanged crystals show the growing-in of a transient band at *ca.* 1700 cm^–1^ (Fig. 20c) with a rate constant of 1/500 ps^–1^ with the back electron transfer occurring with a rate constant of 1/10 ns^–1^.^70^ Importantly, knowledge of the geometry of the binding site allowed assignment of the G_9_ guanine as the site of oxidation. This was based on both the overlap with the nucleobase and the proximity to the metal centre. Interestingly, experiments were also performed on the non-exchanged H_2_O crystals, which revealed the forward electron transfer process to be approximately twice as fast. This isotope effect suggests the possible role of a proton coupled process in the oxidation step, as had been proposed earlier in solution.^63^ The observed lifetimes for the back electron transfers in both cases were similar and not very different from that observed in the solution which suggests the factors controlling this reaction are similar in solution and in the crystal.

## 5. Conclusions and the future

In this review we have focused on the binding, photophysical and photochemical properties of mononuclear complexes of [Ru(LL)_2_dppz]^2+^ bound to double-stranded DNA both in solution and in the crystalline state. The crystal studies have revealed a range of intercalation site geometries and a strong dependence on DNA sequence. The majority of X-ray structures have been solved for the lambda-enantiomers where the TA/TA step appears to be distinctive. The role of the 2-NH_2_ of guanine in determining chromophore orientation from the minor groove is rather well defined for this enantiomer. So far relatively few structures have been determined for the delta enantiomers, and this is certainly an area where more studies are needed, particularly given the importance of this enantiomer for mismatch recognition.

The crystallographic studies also revealed the important role played by the ancillary ligands in determining crystallisation and crystallinity. In particular, for both enantiomers bound to oligonucleotides containing two adjacent guanines, the semi-intercalation of one of the ancillary phen or TAP ligands is a common feature. There is no doubt that the observed semi-intercalation does help stabilise the crystal integrity by cross-linking with a neighbouring duplex when a lambda enantiomer is used, and this could be an important factor in determining the readiness with which the crystals form. For the delta enantiomer, the overall packing is inevitably different, but the binding mode very similar (Fig. 2).

Another important factor to note with the crystal structures is that they involve small DNA molecules (ranging, so far, from 4–12 base-pairs, since longer duplexes typically will not crystallise) so that end-effects are unavoidable. Thus in many of the cases the ‘flipping open’ of the terminal base-pair has been observed permitting base-pairing with the complementary base of another duplex. Another way of looking at this, though, is that the initial driving force for nucleation in crystallisation is actually the adenine–dppz stacking interaction, since the flipping out can also be described as symmetry-related adenine–dppz stacking. The insertion binding mode combines a standard base pairing with the addition of a *syn* adenine forming an additional hydrogen bond and stacking on the phen ligand.

It has been instructive to parallel the crystallographic research on the oligonucleotides systems with photo-physical and photochemical studies in solution. This allows us to investigate the effect of sequence on these photophysical properties, as well as examining whether the intercalation site seen in the crystal is also the preferred location in solution. While photo-luminescence and Raman studies have been widely and successfully used to study the photo-physical properties of some of the complexes in solution, there are extra insights to be gained from transient visible and infrared absorption spectroscopy. In this connection TRIR, by allowing the simultaneous monitoring of the transient species from the metal complex and of the DNA, is of particular value. This technique not only permits the monitoring of the excited states and transient species formed, but can also provide a vibrational spectroscopic imprint of the binding site, as has highlighted the difference of Δ- and Λ-[Ru(phen)_2_dppz]^2+^ to DNA.

Crystallography has already provided insight into the primary processes involved in the DNA oxidation photosensitised by bound [Ru(TAP)_2_dppz]^2+^. These have permitted us to start to understand the factors controlling the yield and rates of reaction in solution and it is interesting that the lambda-complexes show much greater variation in these physical parameters than do their delta isomers, which is perhaps due to more selectivity in binding. However, more structure determinations are required to validate this presumption.

Our understanding of how [Ru(LL)_2_dppz]^2+^ bind to double-stranded DNA opens up the possibility of studying the interaction of the complexes with multi-stranded DNA, such as triplex^72^ and quadruplex.^73^ The potential of the metal complexes to act as probes of such multi-stranded DNA structures is now becoming apparent and it will be fascinating to compare the structures emerging from crystal and NMR studies.

However, it is quite possible that the structures, which dominate in solution, will be different from those selected by crystallisation. For example, it is noteworthy that some of the crystals which are discussed above use oligonucleotides which are known to produce Holliday junctions when crystallised as native DNA, and that the junction form is stabilised by appropriately designed ligands.^74*a*^ The central GGTACC sequence crystallises in the junction form in the presence of Group II cations, whereas for the GGCGCC sequence (as in, *e.g.*, d(TCGCCGCCGA) only does so in some conditions.^74*b*^

So far it has been generally assumed that in solution intercalation of the [Ru(LL)_2_dppz]^2+^ complexes is the overwhelmingly dominant binding mode. Transient spectroscopic investigations of the photophysical/photochemical processes in crystals not only permits the study knowing the precise positioning of the metal complex on the DNA but also may allow an assessment of the importance of other binding modes. In favourable systems (*i.e.* where the quantum yield is high) it should also be possible to monitor the formation of photoproducts, such as adducts formed following electron transfer or photosubstitution. Most photophysical and biophysical studies on the metal complexes and DNA are carried out in relatively dilute solutions, where it is reasonable to expect that secondary binding modes such as semi-intercalation are less important. By contrast the high concentration of the crystals provides great opportunity for such interactions. It should be noted that the concentration regime of the nucleic acid in the nucleus is also much higher, so that it is quite possible that the interactions such as semi-intercalation (which cause kinking of the DNA) could have important biological consequences.

Laser transient spectroscopic absorption spectroscopic methods should be valuable in determining the photoprocesses which proceed in a biological cell and indeed a very recent report by Dietsek's group has just been carried out with [Ru(bpy)_2_dppz]^2+^ in HepG2 cells.^75^ Such studies will provide fundamental information to guide the studies of the rapidly emerging study of ruthenium polypyridyl complexes in biological cells, which should have potential applications such as imaging or photodynamic therapy.^76^
